# Covalently Bonded Ni Sites in Black Phosphorene with Electron Redistribution for Efficient Metal-Lightweighted Water Electrolysis

**DOI:** 10.1007/s40820-024-01331-6

**Published:** 2024-02-14

**Authors:** Wenfang Zhai, Ya Chen, Yaoda Liu, Yuanyuan Ma, Paranthaman Vijayakumar, Yuanbin Qin, Yongquan Qu, Zhengfei Dai

**Affiliations:** 1 State Key Laboratory for Mechanical Behavior of Materials, Xi’an Jiaotong University, Xi’an, 710049 People’s Republic of China; 2 School of Chemistry and Chemical Engineering, Northwestern Polytechnical University, Xi’an, 710072 People’s Republic of China; 3 SSN Research Centre, SSN College of Engineering, Chennai, Tamil Nadu 603110 India

**Keywords:** Black phosphorus, Water electrolysis, Electrocatalyst, Electron redistribution, Covalent functionalization

## Abstract

The metal-lightweighted electrocatalysts for water splitting are highly desired for sustainable and economic hydrogen energy deployments, but challengeable. In this work, a low-content Ni-functionalized approach triggers the high capability of black phosphorene (BP) with hydrogen and oxygen evolution reaction (HER/OER) bifunctionality. Through a facile *in situ* electro-exfoliation route, the ionized Ni sites are covalently functionalized in BP nanosheets with electron redistribution and controllable metal contents. It is found that the as-fabricated Ni-BP electrocatalysts can drive the water splitting with much enhanced HER and OER activities. In 1.0 M KOH electrolyte, the optimized 1.5 wt% Ni-functionalized BP nanosheets have readily achieved low overpotentials of 136 mV for HER and 230 mV for OER at 10 mA cm^−2^. Moreover, the covalently bonding between Ni and P has also strengthened the catalytic stability of the Ni-functionalized BP electrocatalyst, stably delivering the overall water splitting for 50 h at 20 mA cm^−2^. Theoretical calculations have revealed that Ni–P covalent binding can regulate the electronic structure and optimize the reaction energy barrier to improve the catalytic activity effectively. This work confirms that Ni-functionalized BP is a suitable candidate for electrocatalytic overall water splitting, and provides effective strategies for constructing metal-lightweighted economic electrocatalysts.

**Supplementary Information:**

The online version contains supplementary material available at 10.1007/s40820-024-01331-6.

## Highlights

- Black phosphorene (BP) was functionalized by the ionized Ni through an in situ electrochemical exfoliation method.
- Just 1.5 wt% Ni-functionalized BP can stably deliver the efficient alkaline H_2_/O_2_ evolution electrocatalysis.
- Optimized intermediate chemisorption is enabled by Ni–P covalent bonding and electron redistributed surface.

**Supplementary Information:**

The online version contains supplementary material available at 10.1007/s40820-024-01331-6.

## Graphical abstract

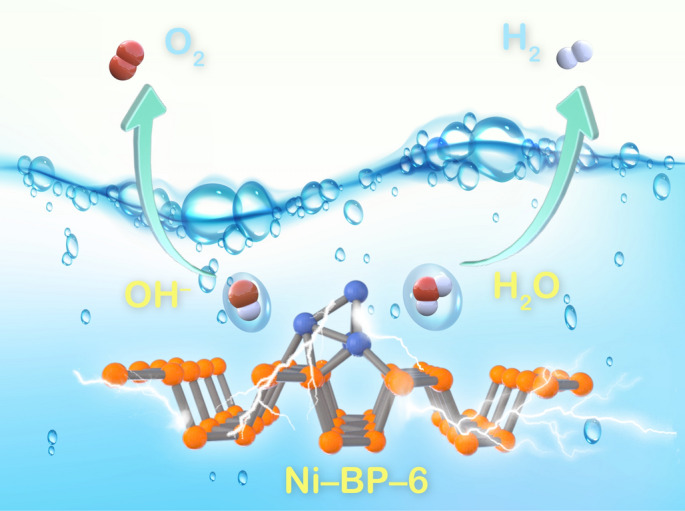

## Introduction

Since the initial publication in 2014, black phosphorus (BP) has sparked considerable attention as an emerging member in two-dimensional (2D) materials [1–6]. Extensive investigations suggest that exfoliated black phosphorus nanosheets (BP NSs, ≤ 10 layers) can be used as the electrocatalysts for oxygen evolution reaction (OER). However, their long-term stability is severely limited due to the presence of the densely packed lone-pair p-electrons (p–e^−^) exposed at the surface [7–9]. The active lone-pair p–e^−^ provides a favorable chemisorption site for O-containing species (OH*, O*, and OOH*). It will also render BP prone to be oxidized, especially when the OER process produces a strong oxidative environment with a high anodic potential [10, 11]. Moreover, the large hydrogen adsorption energy (> 1 eV) makes phosphorene difficult to absorb hydrogen, again limiting its applications in hydrogen evolution reaction (HER) as well as in the overall water splitting (OWS) [12, 13]. Considerable efforts have been devoted to passivating the exposed lone-pair p–e^−^ of BP, typically by covalent functionalization with Lewis acids [10, 14–18]. The formation of covalent bonds leads to the electron redistribution on the catalyst surface and thereafter changes the local electronic structures of active sites through the ligand effect [19, 20]. Especially, the strong coordination bonds between metal species and coordinating atoms with lone-pair electrons like P, S, N, and O can significantly enhance the catalytic stability for long-term application [21–24]. Therefore, these passive covalent functionalization strategies have significant potentials in improving the electrocatalytic performance of BP.

Recently, the 11.3 wt% single-atom Cu bonded BP (Cu–P bond) exhibited a Δ*G*_H*_ close to zero and delivered a high performance for HER beyond single-phase BP electrocatalysts [25]. Due to the strong metal–semiconductor interface effect, the electron transfer from Au nanoparticles to BP layer induced the charge redistribution in the BP/Au heterojunction [14]. More importantly, the addition of Au nanoparticles boosted high adsorption ability of OH^‒^ in 0.1 M KOH, substantially reducing the activation energy required for OER. Generally, the passive covalent functionalization strategy is to develop high-performance electrocatalysts for either HER or OER half-reaction. Rare cases of BP-based materials as bifunctional electrocatalysts can simultaneously deliver both the high HER and OER activities for water splitting. The electron state of catalytic center is the primary consideration to develop the BP-based OWS electrocatalysts, which is highly related to the binding energy of HER/OER adsorbates/intermediates [26–28]. Moreover, sustainable development of future hydrogen economy still relies on the precious-free systems and the high-performance electrocatalysts with low metal contents and cost. A metal-lightweighted electrocatalyst for water splitting is highly desired for sustainable and economic hydrogen energy deployments, but challengeable.

In this work, we reported the BP-based bifunctional OWS electrocatalysts (denoted as Ni-BP-x) by covalently bonding BP NSs with low-content Ni. Polarized P–Ni covalent bonds are demonstrated to enable the electron redistribution across the interfaces, resulting in electron transfer from Ni-to-BP NSs. The manipulated electron acceptor/donor feature confers Ni–BP-x with the decreased rate-determining step (RDS) barrier and enhanced chemical stability. Remarkably, the Ni-BP-6 catalyst with just 1.5 wt% Ni content has presented a high OER/HER activity with small overpotentials of 230/136 mV at 10 mA cm^−2^ in 1.0 M KOH electrolyte, respectively. Especially, the extensive investigations of catalytic mechanism, including *in situ* Raman test and density functional theory (DFT) calculations, have suggested the high activity of Ni-BP-6 originated from the regulated P-Ni coordination environment and optimized electron structure. Importantly, the Ni-BP-6 has delivered the high electrochemical stability for both HER and OER without notable degradation over a period of 50 h. Paring Ni-BP-6 as cathode and anode, the electrocatalysts delivered a low cell voltage of 1.605 V at 10 mA cm^−2^ with excellent stability for overall water splitting. This study provides a preliminary understanding of covalently functionalized BP for bifunctional water splitting electrocatalysis.

## Experimental and Calculations

### Chemical

Bulk BP crystal (99%), nickel foam (99%), nickel chloride (NiCl_2_, > 98%), tetrabutylammonium tetrafluoroborate (TBAB, 98%), acetylene carbon black, 20 wt% Pt/C, ruthenium oxide (RuO_2_, 99%), potassium hydroxide (KOH, 95%), ethanol (99.9%), *N,N*-dimethylformamide (DMF, 99.5%), Nafion (5 wt%) were purchased and used as received. Deionized water (18.2 MΩ cm^−2^) was made using a Millipore Autopure system.

### Preparation of Ni-BP-6

Series of Ni-BP-x were synthesized via electrochemical exfoliation method through a two-phase direct current power supply in the electrolyte, which contained the desired amounts of NiCl_2_ (0.3, 0.6, 0.9 mmol) and TBAB (0.3 g) in DMF (30 mL). The platinum wire and bulk black phosphorus (30 mg) were employed as anode and cathode at 20 V for 30 min to obtain Ni-BP-x. The catalysts were named as Ni-BP-3, Ni-BP-6, and Ni-BP-9, which were corresponding to the electrolytes containing 0.3, 0.6, and 0.9 mmol of NiCl_2_, respectively. Then, Ni-BP-x was transferred to a sealed bottle and treated under the ultrasonication for 3 h. The solids were washed by water and ethanol and then separated by centrifugation until reaching the clean supernatant. Finally, the Ni-BP-x electrocatalysts were dried at 60 ℃ for 12 h under vacuum. BP NSs were synthesized using the method described above for Ni-BP-x in the absence of metal salt in the electrolyte, as well as exfoliation with an applied voltage of 10 V.

### Characterizations

Field-emission scanning electron microscope (FE-SEM, FEI Verios460) and transmission electron microscope (TEM, JEM-2100F) with high-resolution TEM (HRTEM) images, selected area electron diffraction (SAED) patterns, and energy-dispersive X-ray spectrometer (EDS) elemental mapping were adopted to observe the morphologies. X-ray diffraction (XRD, PANalytical X'Pert Pro) with filtered Cu Kα radiation (*λ* = 1.54056 Å) was used to detect the sample’s crystalline structures. X-ray photoelectron spectroscopy (XPS, Thermo Fisher Scientific ESCALAB Xi +) was used to obtain the electronic states and the elemental composition near the catalyst surface. Fitting analysis of XPS data using XPSPEAK41 software. *Ex* situ Raman spectra were probed using Horiba HR800 spectrometer with excitation source of 532 nm laser. *In situ* Raman spectra was probed using thermo scientific DXR3xir Raman Imaging Microscope with excitation source of 532 nm laser. Ethanol dilutions of the catalyst were added dropwise to the mica sheets, and an atom force microscope (AFM, DIMENSION IOON) was employed to investigate the thickness of the exfoliated black phosphorus nanosheets by testing in an air environment. Element analysis of P and Ni of catalyst was detected by ICP-MS (NexION 350D).

### Electrochemical Measurements

All electrochemical measurements were executed with a three-electrode configuration on a CHI 650E Electrochemical Workstation (Shanghai Chenhua, China) in 1.0 M KOH solution. Graphite rods and Hg/HgO were used as the counter electrode and reference electrode, respectively. Catalysts (4 mg) and acetylene carbon black (1 mg) were mixed with ethanol (300 μL), ultrapure water (170 μL), and 5 wt% Nafion (30 μL) through sonication for 2 h. Afterward, the catalytic ink was dripped on nickel foam (NF, 0.25 cm^2^) work electrode. For OER and HER, the Ni loadings on the electrode of Ni-BP-3, Ni-BP-6, and Ni-BP-9 are 8.2, 9.6, and 8 µg, respectively. The potential in this article was converted to RHE using *E*_vs. RHE_ = *E*_vs. Hg/HgO_ + 0.059pH + 0.098 V (at room temperature). Linear sweep voltammetry (LSV) curves were examined with a scanning speed of 5 mV s^−1^ and potential ranges of 1.2–1.8 V (vs RHE) for OER and − 0.6–0.1 V (vs RHE) for HER, respectively. Electrochemical impedance spectroscopy (EIS) measurements at a certain potential (potential of the samples at 10 mA cm^−2^) with a frequency from 0.1 Hz to 100 kHz. The electrochemically active surface area (ECSA) measurements within the range of 1.014–1.114 V (vs. RHE) for OER and − 0.046–0.054 V (vs. RHE) for HER were conducted at various scan rates (20, 40, 60, 80, 100, and 120 mV s^−1^). *C*_dl_ was derived from the plot of the Δ*J* = (*J*_+_  − *J*_−_)/2 at 1.064/0.004 V versus RHE against the sweep rates. Long time stability for OER, HER, and overall water splitting were measured using chronopotentiometry method with applied potentials of 1.474, − 0.190, and 1.658 V vs. RHE, respectively.

### Electrical Humidity Sensing Test

The catalytic ink was dipped on a Au sensing electrode (200 µm spacing) and connected with a TO-5-case to fabricate a sensor. The humidity sensing properties were tested in a home-built dynamic sensing system with gas flow controllers (500 sccm). The different humidity was generated by mixing the dry air and wet air (from the saturated K_2_SO_4_ solution, 60%RH). The electrical resistance of sensing device was measured by Keithley DMM6500 digital multimeter. The humidity response was calculated by the value of *R*_humid_/*R*_air_, where *R*_air_ and *R*_humid_ were the sensor resistances under air and humid ambient.

## Results and Discussion

### Synthesis and Characterization of Ni-BP-x

**Fig. 1:**
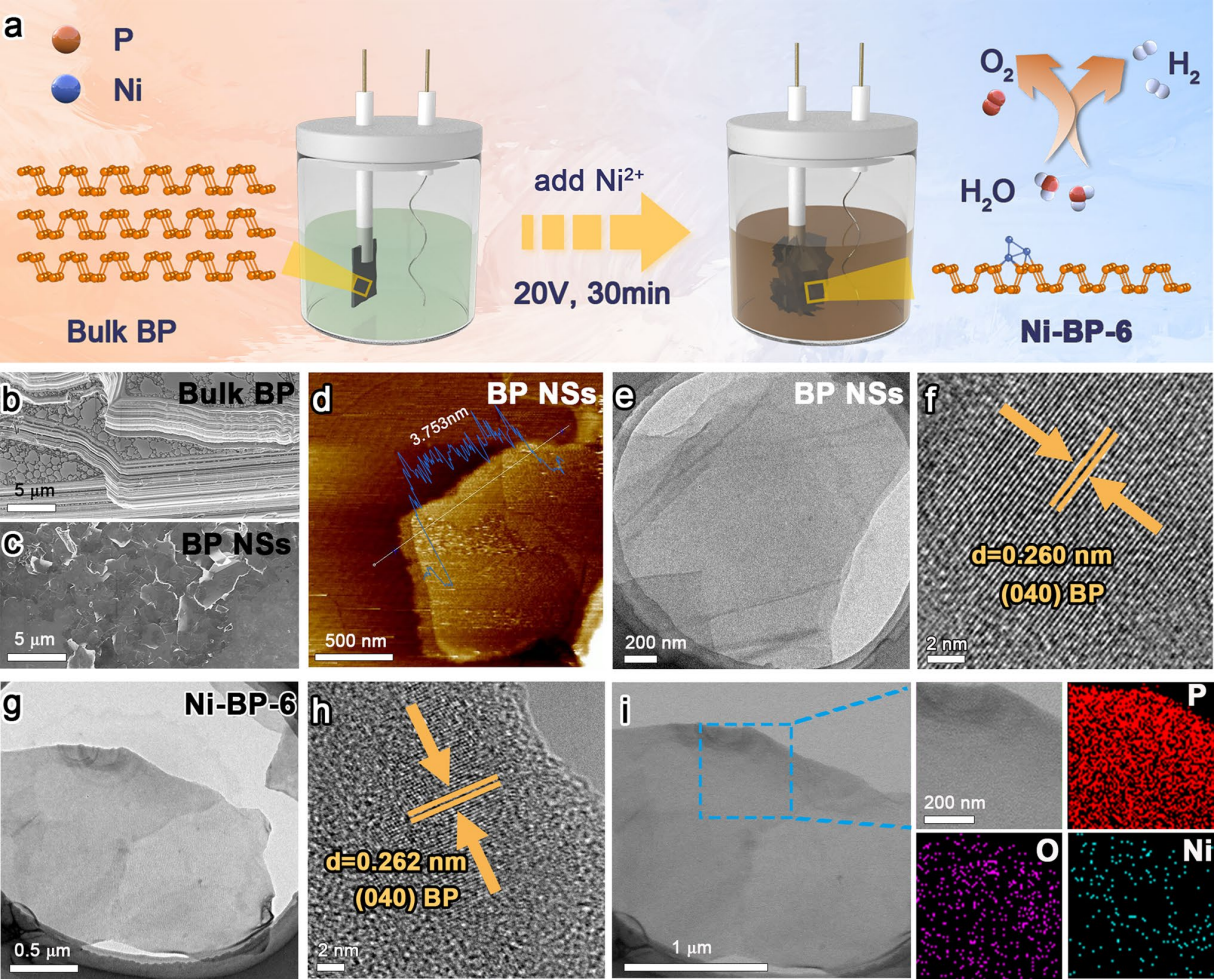
Synthesis method and microstructures for Ni-functionalized BP. **a** Synthetic scheme for Ni-BP-6. **b** SEM image of bulk BP. Characterizations of BP NSs: **c** SEM, **d** AFM, **e** TEM, and **f** HRTEM images. TEM characterizations of Ni-BP-6: **g** TEM, **h** HRTEM, and **i** EDS mapping images

As illustrated in Fig. 1a, the Ni-BP-x electrocatalysts were prepared via the one-step electro-exfoliation/covalent functionalized method from bulk BP and NiCl_2_. Similarly, BP NSs were synthesized from bulk BP without the addition of metal ions (Fig. S1). The FE-SEM image revealed the ordered multilayer lamellar structure of bulk BP (Fig. 1b), which were further exfoliated into few-layer nanosheets after the treatments (Fig. 1c) [29]. As revealed from AFM image in Fig. 1d, the thickness of BP NSs is *ca.* 3.75 nm (7 layers, the single layer thickness is 0.53 nm) [30]. After the metal covalent functionalization, the BP NSs remained as a micron-sized nanosheet-like structure, suggesting that the macroscopic morphology of the structure was not damaged (Fig. S2). X-ray diffraction (PXRD) patterns proved that the Ni-BP-x samples were well-crystallized, with no obvious diffraction peaks of Ni metal phase (Fig. S3). All the diffraction peaks of Ni-BP-x can be indexed to the BP (JCPDS No. 01-073-1358) [31–33]. A series of peaks located at 17.2°, 34.5°, and 52.6° can be assigned to the (020), (040), and (060) planes of BP NSs, respectively. The peak positions of Ni-BP-3, Ni-BP-6, and Ni-BP-9 materials slightly shifted to lower angles in comparison with those of BP NSs. According to the Bragg’s law, the stretched crystal plane spacing of BP NSs is caused by the covalent bonding of Ni. The few-layer BP NSs successfully incorporate Ni atoms as evidenced by the lattice expansion and lack of metal phase, which is consistent with the subsequent HRTEM data. TEM analysis revealed a comparatively thin view of BP NSs (Fig. 1e) with a well-defined fringe spacing of 0.260 nm, corresponding to the (040) crystal planes in the high-resolution TEM (HRTEM) image (Fig. 1f). The SAED pattern shows the rings corresponding to the (111), (112), and (002) crystal planes of BP NSs (Fig. S4). As demonstrated in Fig. 1g, the Ni-BP-6 maintained the lamellar microstructure after the introduction of Ni^2+^ ions. The SAED pattern of Ni-BP-6 material presents the (111), (221), and (022) crystal planes of BP NSs (Fig. S5) in the absence of any diffraction rings of Ni metal phase, indicating the Ni dispersion on the BP NSs. The HRTEM image exhibits the (040) crystal plane with a fringe spacing of 0.262 nm (Fig. 1h). Such a lattice after Ni inserting/covalent binding is slightly larger than that of BP NSs (0.260 nm), which demonstrates that the Ni element was covalent functionalized with BP. In the Ni-BP-6 sample, the elemental mapping images have clearly illustrated the uniform distribution of P/Ni/O (Fig. 1i). Ni-BP-3 and Ni-BP-9 exhibit the preserved lamellar structure of BP NSs, with slight lattice stretching and uniform component distribution (Figs. S6–S7). The Ni inserting/covalent amounts of Ni-BP-3, Ni-BP-6, and Ni-BP-9 were 0.7, 1.5, and 2.0 wt%, respectively, which were determined by inductively coupled Plasma mass spectrometer (ICP-MS) examinations (Table S1).

**Fig. 2:**
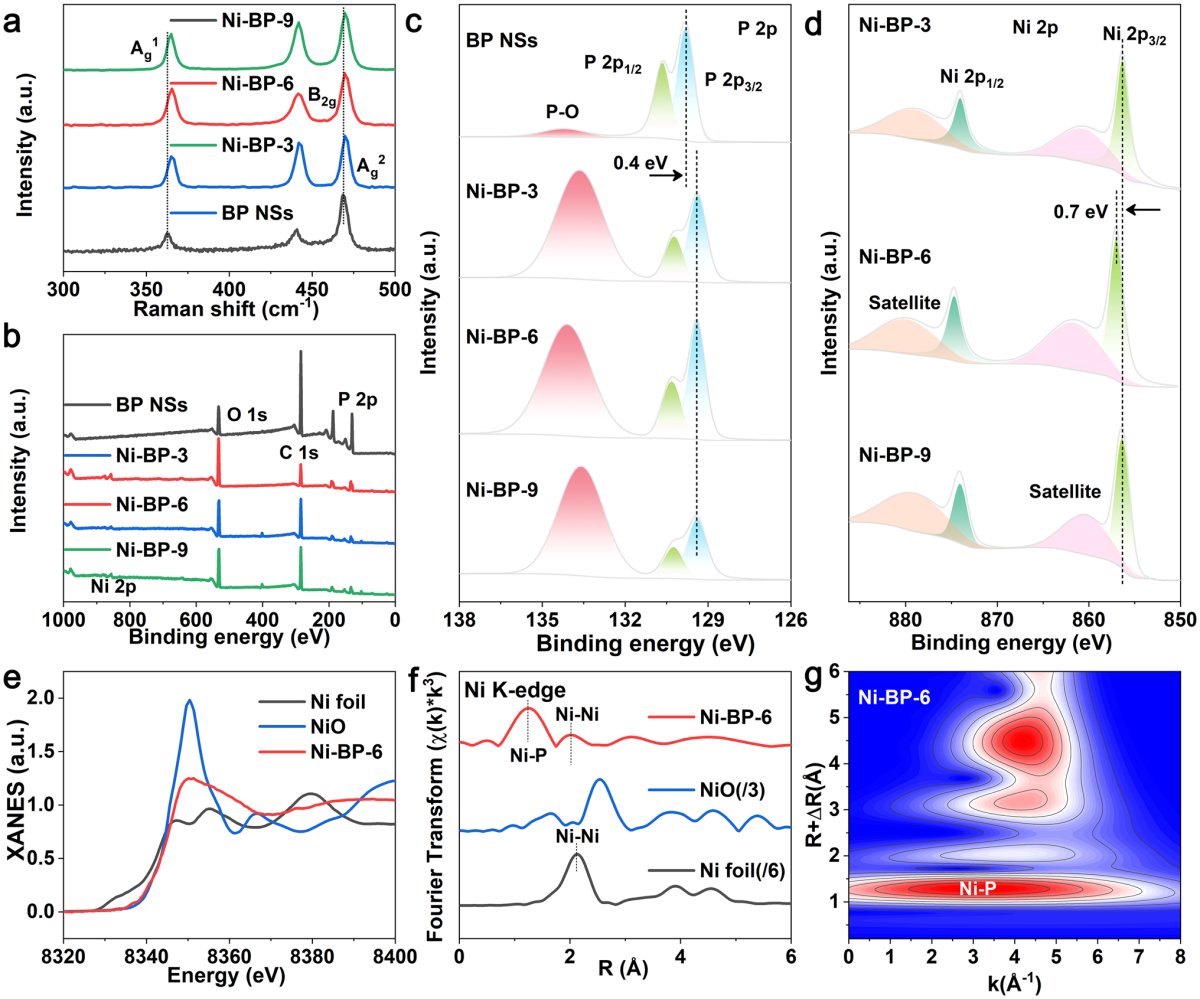
Chemical states and XANES analyses of Ni-functionalized BP. **a** Raman spectra. **b** XPS survey of samples. **c** P 2*p* XPS profiles of BP NSs, Ni-BP-3, Ni-BP-6, and Ni-BP-9. **d** Ni 2*p* XPS profiles of BP NSs, Ni-BP-3, Ni-BP-6, and Ni-BP-9. **e** XANES spectra for Ni-BP-6, NiO, and Ni foil. **f** Ni K-edge EXAFS spectra in R space. **g** Wavelet transform for the k^3^-weighted EXAFS signal for Ni-BP-6

The variations in the Raman spectra of different samples were further compared in Fig. 2a. Three characteristic peaks of BP NSs of *A*_g_^1^ (362.65 cm^−1^, out of plane), *A*_g_^2^ (469.13 cm^−1^, in-plane armchair), and *B*_2g_ (440.54 cm^−1^, in-plane zigzag) were observed [11]. Comparatively, the Raman peaks of Ni-BP-3, Ni-BP-6, and Ni-BP-9 blue-shifted to the higher wavenumbers, suggesting the intimately covalent interaction between metal ions and BP [29]. Additionally, Fig. S8 depicts the FTIR spectra of samples, demonstrating the vibration of the P–O, P=O, and O–H bonds [34, 35]. Surface chemical and electronic states of various electrocatalysts were further explored by XPS. The XPS survey scan spectra of the samples manifest the coexistence of P and Ni elements (Fig. 2b). High-resolution XPS spectra of F 1*s* and B 1*s* (Figs. S9, S10) disclose that no heteroatoms (F and B) were introduced during the exfoliation process. High-resolution P 2*p* XPS spectra (Fig. 2c) of BP NSs could be deconvoluted into three peaks at 130.65 eV (P 2*p*_1/2_), 129.8 eV (P 2*p*_3/2_), and 134.2 eV (P–O species). In contrast to BP NSs, the P 2*p*_1/2_ and P 2*p*_3/2_ peaks of the Ni-BP-3, Ni-BP-6, and Ni-BP-9 samples showed a modest -0.4 eV shift (Fig. 2c and Table S2), highlighting BP NSs as an electron acceptor in the Ni-functionalized BP structures [25]. In the high-resolution Ni 2*p* spectra (Fig. 2d and Table S3), the peaks at the binding energies of 874.0 and 856.3 eV correspond to the Ni 2*p*_1/2_ and Ni 2*p*_3/2_ levels of Ni^2+^ [36, 37]. It could be seen that the Ni 2*p* peaks of Ni-BP-6 (874.7 eV, Ni 2*p*_1/2_; 857.0 eV, Ni 2*p*_3/2_) have shifted toward higher binding energies (about 0.7 eV) compared to Ni-BP-3 and Ni-BP-9 (874.0 eV, Ni 2*p*_1/2_; 856.3 eV, Ni 2*p*_3/2_). This indicates that Ni of Ni-BP-6 exhibits stronger interaction with BP NSs and favorable electron transfer [38]. Given that a higher valence state of Ni is frequently thought to be the highly active site in catalytic process with the reduced reaction energy, the trend toward high-valence Ni in Ni-BP-6 will predict well for its superior water oxidation performance [39, 40].

X-ray absorption near-edge structure (XANES) and extended X-ray adsorption fine structure (EXAFS) spectroscopy were used to recognize the detailed structural information of the Ni-BP-6 catalyst. Figure 2e shows the Ni K-edge XANES spectra of Ni-BP-6, NiO, and Ni foil. The absorption edge (8340–8346 eV in XANES spectra) of Ni-BP-6 is very close to that of NiO, further unveiling a similar electronic structure (Ni^2+^) [41, 42]. The Fourier-transformed (FT) k^3^-weighted EXAFS spectra of the R space for the Ni-BP-6 exhibit Ni–P bond and Ni–Ni bond (Fig. 2f) [43]. Ni *K*-edge EXAFS and the curvefit results of the R space (FT magnitude and imaginary component) are shown in Fig. S11. The fitting results agree well with the Ni-BP-6 model in the DFT calculation. Detailed metals *K*-edge EXAFS fitting parameters can be found in Table S4. Since the characteristic peaks of the Ni–P bond in Fig. 2f are located at similar positions to the Ni–O bond, the wavelet transform (WT) of the Ni K-edge EXAFS oscillations were further analyzed. The results have confirmed that the backscattered atoms to the Ni are indeed not O atom but P atom, because the WT-EXAFS analysis can provide resolution in R and k spaces (Figs. 2g and S12) [44]. The above results have indicated the present of Ni–P coordination.

**Fig. 3:**
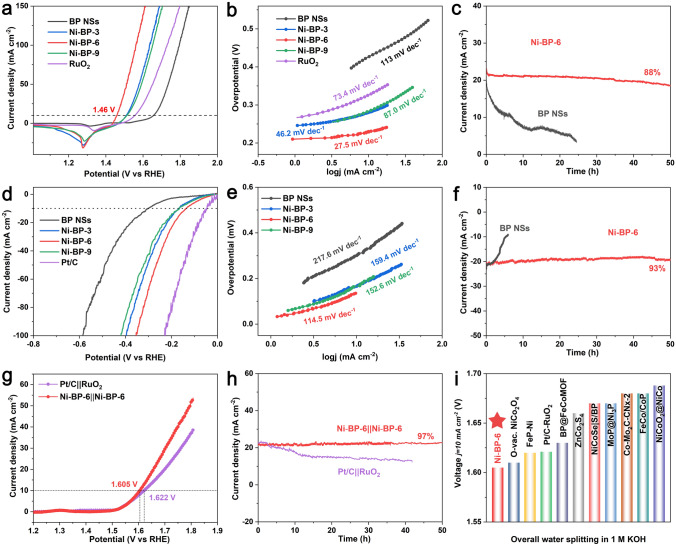
Electrochemical performances of Ni-BP-6 at 1.0 M KOH.** a** OER LSV curves, **b** Corresponding Tafel plots. **c** OER *i-t* curve. **d** HER LSV curves, **e** Corresponding Tafel plots. **f** HER *i-t* curve. **g** Polarization curves of overall water splitting (OWS) based on Ni-BP-6||Ni-BP-6 and Pt/C||RuO_2_ couples. **h** Catalyst durability for OWS for 50 h. **i** Comparison of the OWS voltages at 10 mA cm^−2^ with recently reported OWS bifunctional catalysts

To assess electrocatalytic performance, various catalysts electrodes were evaluated by using a standard three-electrode system at 1.0 M KOH. The OER performances of the Ni-functionalized BP catalysts with different molar ratios of Ni are shown in Fig. 3a. Ni-BP-6 exhibits a superior OER activity with an overpotential (*η*_10_) of 230 mV at 10 mA cm^−2^, compared to those η_10_ values of BP NSs (427 mV), Ni-BP-3 (281 mV), Ni-BP-9 (284 mV), and RuO_2_ (408 mV) (Fig. S13). The corresponding OER kinetics were compared from their Tafel slope values (Fig. 3b). Ni-BP-6 still exhibited the faster OER kinetic with a lower Tafel slop of 27.5 mV dec^−1^ than those of BP NSs (113 mV dec^−1^), Ni-BP-3 (46.2 mV dec^−1^), Ni-BP-9 (87 mV dec^−1^), and RuO_2_ (73.4 mV dec^−1^). The electrochemical impedance spectra (EIS) investigations (Fig. S14) showed the similar charge transfer resistance (*R*_ct_) of Ni-BP-3, Ni-BP-6, and Ni-BP-9. This indicates that the difference in OER activity is not caused by impedance but by the inherent activity of the materials themselves [45]. To further explore the intrinsic activity, LSV curves of OER catalysts were normalized by Ni loading (Fig. S15), and Ni-BP-6 still exhibited the highest OER activity. Additionally, the linear slope of the double layer capacitance (*C*_dl_) was used to interpret the electrochemical active area (ECSA), which was determined from the CV curves of each catalyst at varied scanning speeds (Fig. S16). The *C*_dl_ value of Ni-BP-6 was 9.7 mF cm^−2^, compared to those *C*_dl_ of BP NSs (7.7 mF cm^−2^), Ni-BP-3 (11.2 mF cm^−2^), and Ni-BP-9 (8.8 mF cm^−2^) (Fig. S16e**)**. It suggests that Ni-BP-6 has a relatively high intrinsic activity, which is consistent with the findings shown in the aforementioned impedance data (Fig. S14) [46]. The ECSA-normalized polarization curves (*j*_ECSA_) in Fig. S17 display that the intrinsic activity of Ni-BP-6 is still much higher than that of other BP-based catalysts. The chronopotentiometry curve has indicated that Ni-BP-6 operated steadily for 50 h at an applied voltage of 1.474 V with 88% current density retention (Fig. 3c).

The HER characteristics in 1.0 M KOH are shown in Figs. 3d–f and S18–S21. Figure 3d presents the LSV curves of various catalysts. The Ni-BP-6 has delivered the lowest *η*_10_ (136 mV) in comparison with BP NSs (301 mV), Ni-BP-3 (166 mV), Ni-BP-9 (167 mV), and Pt/C (46 mV) (Fig. S18). Ni-BP-6 also showed the faster HER kinetic with a lower Tafel slope of 114.5 mV dec^−1^ than those of BP NSs (217.6 mV dec^−1^) and other BP-based samples as well (Fig. 3e). Ni-BP-6 has also exhibited the best intrinsic activity when the HER LSV curve is normalized to the Ni loading (Fig. S19). Although Ni-BP-3 has the largest ESCA, the Ni-BP-6 catalysts still demonstrates the highest intrinsic activity in terms of the ECSA-normalized current density (Figs. S20, S21) [45, 46]. The *i−t* stability testing of Ni-BP-6 has shown that the current retention was 93% after 50 h (Fig. 3f). In view of the excellent HER and OER performances, we assembled Ni-BP-6 as cathode and anode to access the overall water splitting (OWS) performance in 1.0 M KOH electrolyte (Fig. 3g). The LSV curve of Ni-BP-6||Ni-BP-6 has showed that the potential at 10 mA cm^−2^ (1.605 V) was lower than the commercial Pt/C||RuO_2_ pair (1.621 V). The *i−t* test in Fig. 3h further demonstrates that the Ni-BP-6||Ni-BP-6 couple delivered the outstanding stability in OWS, maintaining ~ 97% activity after 50 h continuous operation. The stable catalytic performance can be also reflected from the slight P and Ni dissolution during the OER process (Table S5). Figure 3i and Table S6 present a comparison of the competitive OWS performance of Ni-BP-6||Ni-BP-6 with other reported OWS catalysts. Further, it is also found that the trace amount of Fe impurities in the KOH electrolyte presents a very slight influence on the η_10_ potentials (Figs. S22, S23 and Table S7).

**Fig. 4:**
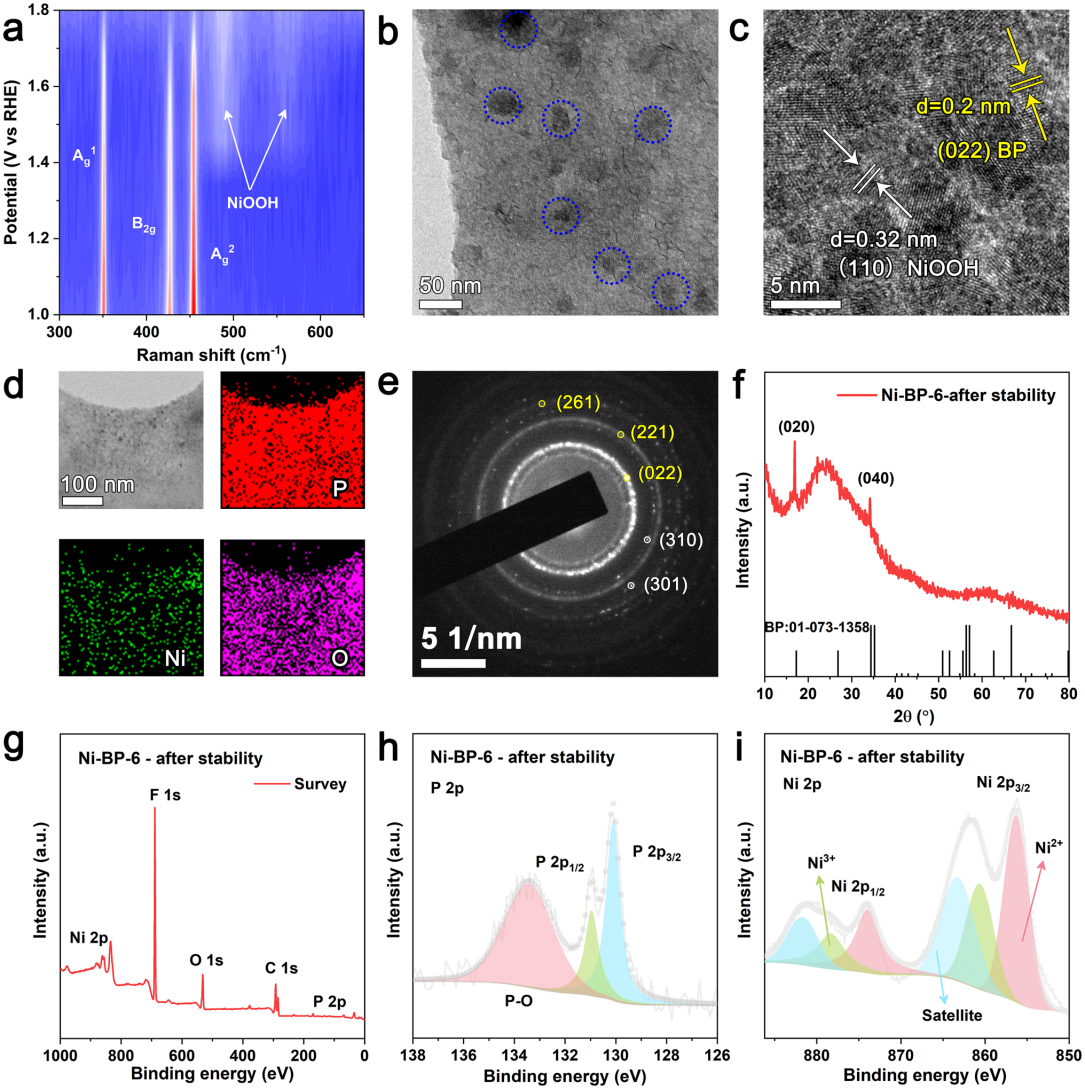
**a** Postmortem structural analyses after stability tests. **a** 2D *in situ* Raman intensity map of Ni-BP-6. Characterizations of Ni-BP-6 after stability test: **b** TEM, **c** HRTEM, **d** EDS mapping. **e** SAED pattern, **f** XRD pattern, **g** XPS surveys, **h** P 2*p* XPS profile, and **i** Ni 2*p* XPS profile of Ni-BP-6 after stability test

Surface reconstruction (SRC) is a typical phenomenon in water splitting reactions, notably in the OER process, for the Ni or Co-based electrocatalysts [47, 48]. To authenticate this SRC behavior, we evaluated Ni-BP-6 during the OER using an *in situ* Raman instrument (Fig. S24). In the initial stage of OER, the *in situ* Raman spectrum showed three characteristic peaks of BP NSs (*A*_g_^1^, *A*_g_^2^, and *B*_2g_). As the OER reaction progressed, two distinct Raman peaks appeared at 475 and 556 cm^−1^, which are the representative band couples of *γ*-NiOOH (Fig. 4a) [49]. These findings clearly indicate an oxidation process from the initial Ni^2+^ to the final NiOOH during OER cycling, which are served as the real active site for continuous cycles. This phenomenon was further verified by TEM and XPS observations of Ni-BP-6 after stability testing. As shown in Fig. 4b, the spent Ni-BP-6 electrocatalyst has remained the sheet-like structure. The darker part circled in blue was the carbon during configuring the catalyst ink. The well-defined fringe spacing was 0.2 nm corresponding to the (022) crystal plane of BP (Fig. 4c). The sample also showed the NiOOH (110) crystalline plane with a fringe spacing of 0.32 nm. In accordance with a consistent elemental distribution, EDS mapping showed the uniform distribution of Ni and P elements (Fig. 4d). The SAED pattern of the Ni-BP-6 electrocatalysts revealed the (022), (221), (261) crystal planes of BP, as well as the (310), (301) crystal planes of NiOOH (Fig. 4e). The new NiOOH phase could not be observed in the XRD pattern due to the low Ni content in the Ni-BP-6 sample (Fig. 4f).

XPS was employed to analyze the valence state changes of elements so as to confirm the surface reconstruction phenomena of Ni-BP-6 (Fig. 4g–i). Since the usage of Nafion and the difficulty for the complete removal of Nafion, the F 1* s* could be seen in the whole XPS spectrum of the spent electrocatalysts (Fig. 4g). As shown in Fig. 4i, the high-resolution Ni 2*p* XPS spectra present the Ni^3+^ peaks at 878.3 eV (Ni 2*p*_1/2_) and 860.6 eV (Ni 2*p*_3/2_) as well as the Ni^2+^ peaks at 873.96 eV (Ni 2*p*_1/2_) and 856.26 eV (Ni 2*p*_3/2_). It could assume that the Ni sites are critical for maintaining the OER catalytic performance, and the Ni^2+^ to Ni^3+^ conversion occurs during the cycle stage, greatly contributing to the decline in catalyst overpotential [39, 50].

**Fig. 5:**
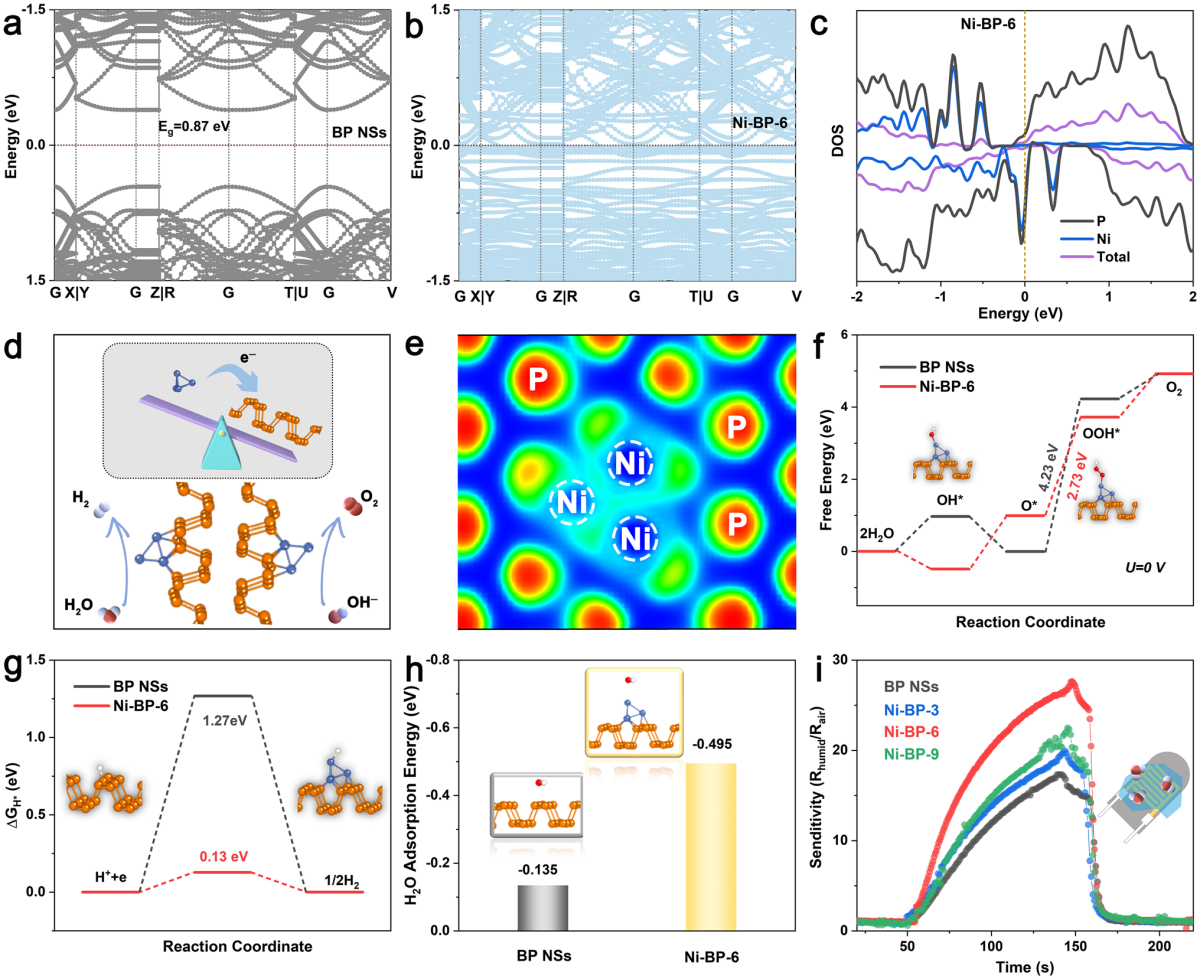
Theoretical profiles for the BP NSs and Ni-BP-6 structures. The band structure of **a** BP NSs, **b** Ni-BP-6. **c** DOS of Ni-BP-6. **d** Schematic work function diagram (top) of BP NSs and Ni_4_. And proposed OER and HER process (bottom) of Ni-BP-6. The brown and blue balls represent P and Ni atoms. **e** The electron localization function mapping on the (001) surface of Ni-BP-6. The red/blue colors refer to positive/negative charged areas, respectively. **f** The free energy diagram of the OER process on BP NSs and Ni-BP-6 at *U* = 0 V. Insets show the optimized structures with the adsorption of oxygen-containing intermediates during OER process of Ni-BP-6. **g** Hydrogen adsorption Gibbs free energy of different catalysts. The insets from left to right show the BP NSs and Ni-BP-6 with the adsorption of hydrogen. **h** H_2_O adsorption model and energy of BP NSs and Ni-BP-6. The insets from left to right show the BP NSs and Ni-BP-6 with the adsorption of H_2_O. **i** Humidity sensing performances of BP NSs, Ni-BP-3, Ni-BP-6, and Ni-BP-9 at 60%RH. Inset is the sensor electrode structure

Ni-functionalized BP was theoretically profiled and analyzed by DFT calculations on Device Studio [51]. For the simplicity of calculations, the typical Ni_4_ incorporated structures were constructed and compared with bare BP (Fig. S25). Figure 5a, b represents the band structures of BP NSs and Ni-BP-6 with bandgaps (*E*_g_) of 0.87 and 0 eV, respectively. The decreased *E*_g_ suggests a significant enhancement of electrical conductivity after the Ni functionalization. The density of states (DOS) in Figs. 5c and S26 illustrated the typical semiconductor characteristics of BP, while Ni-BP-6 displayed a certain electronic state distribution near the E_F_ with the favorable electrical conductivity [52]. Furthermore, the work functions and Ni-BP electron transfer were also calculated and presented in Fig. 5d. The work functions (*Φ*) of BP NSs and Ni-BP-6 are 5.02 and 4.126 eV, respectively (Fig. S27). The *Φ* results indicate the Ni-to-BP electron transfer pathway and the electron deficient state of Ni, as consistent as the XPS results (Fig. 2c, d). The electron localization function (ELF) analysis (Fig. 5e) has further verified the redistribution of charge density. Figure S28 displays the overall OER stages on the surface of BP NSs and Ni-BP-6.

Figure 5f presents the free energy data of BP NSs and Ni-BP-6 at *U* = 0 during the OER process. The rate-determining step (RDS) for bare BP is the formation of OOH* (4.23 eV) in agreement with the previous reports [10, 53, 54]. While for Ni-BP-6, the Ni site shows a profoundly smaller RDS barrier (2.73 eV) than BP (4.23 eV), reflecting an accelerated conversion from O* to OOH* and BP-beyond OER kinetics. Detailed information on the stable adsorption energies of the OER process at 1.23 V is shown in Fig. S29 and Tables S8, S9. With regard to HER, the adsorption energy of H (Δ*G*_H*_ descriptor) was calculated for bare BP NSs and Ni-BP-6 (Fig. 5g). The Δ*G*_H*_ value of BP is 1.27 eV, showing an inferior H adsorption energy. Ni-BP-6 possesses a near-natural Δ*G*_H*_ value (0.13 eV) and has the most energetic HER activity (Table S10). Moreover, for alkaline HER, the H_2_O dissociation energy to form the H* intermediate can assess the activity and kinetic of catalysts. As demonstrated in Fig. S30, the dissociation barrier on the Ni-BP-6 surface is dramatically reduced from 2.14 eV (BP) to 1.67 eV, showing the increased H_2_O dissociation and H* production kinetics. Since the initial step for water electrolysis is the water absorption on the catalyst surface, we conducted the H_2_O adsorption energy calculations (Fig. 5h) and humidity sensing (Fig. 5i) for these materials to understand the catalytic reaction mechanism [29]. As shown in Figs. 5h and S31, the Ni-BP-6 (− 0.495 eV) has showed higher H_2_O adsorption energy (Δ*G*_H2O_) than pure BP (− 0.135 eV), Ni-BP-3 (− 0.278 eV), and Ni-BP-9 (− 0.354 eV). Detailed calculation values are shown in Table S11. The higher water adsorption capacity of the Ni-BP-6 structure can facilitate the following steps of water electrolysis. Such an improved interaction of Ni-BP-6 with water was also studied and evidenced by the humidity sensing tests in Fig. 5i, where the Ni-BP-6 sample displayed a higher humidity sensing response. The optical contact angle measurements have elucidated that Ni-BP-6 featured more enjoyable hydrophilic surface in comparison with the bare BP NSs, Ni-BP-3, and Ni-BP-9 (Fig. S32). Since the XPS results suggest the surface oxidation on the BP NSs and Ni-BP-6, we have also conducted the DFT calculations for the surface-oxidized BP (O-BP) and Ni-BP-6 structures (O-Ni-BP-6) for OER (Fig. S33). The surface-oxidized structures show higher electronic state distribution near the E_F_ with rapid electron transfer compared to the unoxidized ones (Fig. S33b–e). It can be seen that the O-Ni-BP-6 still exhibits the more balanced energy barriers than O-BP in terms of OER free energy, Δ*G*_H*_, and Δ*G*_H2O_ (Fig. S33f–i). All the above results indicate that the metallic incorporation of BP modulates the electronic structure and promotes the OER and HER kinetics.

## Conclusions

In conclusion, low-content Ni functionalization is developed as a covalent modification technique for BP NSs, resulting in considerable improvements in OER/HER/OWS performance. On the basis of experimental results and theoretical calculations, the covalently bonded P-Ni can regulate the electronic structure and electron redistribution, optimize the reaction energy barrier, and strengthen the catalyst stability. The optimized Ni-functionalized BP materials with 1.5 wt% metal content have presented excellent and stable catalytic performances with low *η*_10_ overpotentials in HER (136 mV) and OER (230 mV) of Ni-BP-6 at 10 mA cm^−2^ under 1.0 M KOH electrolytes. Importantly, they both maintain no obvious decay at 20 mA cm^−2^ for 50 h. By integrating Ni-BP-6 as the cathode and anode, overall water splitting can be achieved at a low cell voltage of 1.605 V at 10 mA cm^−2^ for 50 h, benchmarking the BP-based OWS electrocatalysts. It is also well interpreted by the reaction step energy barrier determined by DFT calculations with the decreased RDS step energy barriers. *In situ* Raman studies and electrochemical measurements have revealed how the Ni-functionalized BP structures behaved during OER processes in terms of surface reconstruction (SRC). This study carries interesting potentials for designing chemical functionalized BP electrocatalysts for economic, efficient, and stable water splitting applications.

## Supplementary Information

Below is the link to the electronic supplementary material.

Supplementary file 1 (PDF 3071 kb)
