# Supplementary material for: Covalently Bonded Ni Sites in Black Phosphorene with Electron Redistribution for Efficient Metal-Lightweighted Water Electrolysis
Source: Nanomicro Lett. 2024 Feb 14;16:115. doi: 10.1007/s40820-024-01331-6 (PMC10866855; doi:10.1007/s40820-024-01331-6)
Supplement: Supplementary file 1 — (PDF 3071 kb) [file 40820_2024_1331_MOESM1_ESM.pdf]

Supporting Information for

Covalently Bonded Ni Sites in Black Phosphorene with Electron Redistribution for Efficient Metal-Lightweighted Water Electrolysis

Wenfang Zhai[1], Ya Chen[1], Yaoda Liu[1], Yuanyuan Ma[2], Paranthaman Vijayakumar[3] Yuanbin Qin[1], Yongquan Qu[2,*] and Zhengfei Dai[1,*]

[1]State Key Laboratory for Mechanical Behavior of Materials, Xi'an Jiaotong University, Xi'an 710049, P. R. China

[2]School of Chemistry and Chemical Engineering, Northwestern Polytechnical University, Xi'an, 710072, P. R. China

[3]SSN Research Centre, SSN College of Engineering, Tamil Nadu 603110, India

*Corresponding authors. E-mail: sensdai@mail.xjtu.edu.cn (Z. D.); yongquan@nwpu.edu.cn (Y. Q.)

Note S1 Density Functional Theory Calculation

DFT was used to study the reaction energy barriers of BP and Ni-BP-6 during reaction process for overall water splitting. Device Studio program provides a number of functions for performing visualization and modeling [S1].The DFT calculations were carried out based on projector augmented-wave (PAW) method and the Vienna Ab Initio Simulation Package (VASP) code, employing Perdew-Burke-Ernzerhof (PBE) and generalized gradient approximation (GGA) [S2-S4]. A 6×4×1 supercell of black phosphorus (0 2 0) was used k-points grid of 3×3×1 and 6×6×1 for structure optimization and density of states (DOS) calculations, respectively [S5]. The cutoff energy was 450 eV and the convergence of energy and forces were 10^{-5} eV and 0.01 eV $Å^{-1}$.

S1.1 Density Functional Theory Calculation of OER Progress

In an alkaline environment, OER calculation could occur in the following four-electron pathways:

$$OH^- + {}^* \leftrightarrow {}^*OH + e^- \quad (S1)$$

$$^*OH + OH^- \leftrightarrow {}^*O + H_2O + e^- \quad (S2)$$

$$^*O + OH^- \leftrightarrow {}^*OOH + e^- \quad (S3)$$

$$^*OOH \leftrightarrow O_2 + {}^* + H_2O + e^- \quad (S4)$$

where * represents the active site on the surface, the adsorption energy for adsorbates ΔE_{ads} for OOH*, O*, and OH* can be calculated by the following equations:

$$\Delta EO^* = E(O^*) - E(^*) - (EH_2O - EH_2) \quad (S5)$$

$$\Delta EOH^* = E(OH^*) - E(^*) - (EH_2O - 1/2EH_2) \quad (S6)$$

$$\Delta EOOH^* = E(OOH^*) - E(^*) - (2EH_2O - 3/2EH_2) \quad (S7)$$

The adsorption free energy for adsorbates ΔGads can be calculated by the following equation:

$$\Delta G_{ads} = \Delta E_{ads} + \Delta ZPE - T\Delta S \quad (S8)$$

ΔZPE-TΔS is used as energy correction values, and the results of the corresponding steps are listed in Tables S5-S6.

S1.2 Density Functional Theory Calculation of HER Progress

The free energy of hydrogen adsorption (ΔG_{H*}) is defined as

$$\Delta G_{H*} = \Delta E_{H*} + \Delta E_{ZPE} - T\Delta S_H \quad \text{(S9)}$$

Where ΔE_{H*} is the adsorption energy of hydrogen, ΔE_{ZPE} is the zero-point energy, T is for temperature and ΔS_H is the entropy differences of H between the adsorbed hydrogen and gas phase hydrogen (H_2). The H* denotes the catalytic active site for hydrogen adsorption. ΔE_{H*} is defined as

$$\Delta E_{H*} = E(Surf + H^*) - E(Surf) - 1/2E(H_2) \quad \text{(S10)}$$

Where E(Surf+H*), E(Surf) and E(H_2) represent total energies of the electrocatalyst with adsorbed hydrogen atom, the catalyst itself and H_2, respectively.

Under standard conditions, the value of ΔE_{ZPE}-$T\Delta S_H$ is chosed as the reference. Thus, equation (S9) can be abbreviated as equation (S11)

$$\Delta G_{H*} = \Delta E_{H*} + 0.24 \quad \text{(S11)}$$

In order to match the experimental and theoretical calculations more closely, H atoms adsorbed on the surface of BP and Ni-BP-6 were used to calculate their (ΔG_{H*}). The detailed data of (ΔG_{H*}) of electrocatalyst can be found in Table S7.

Note S2 XAFS Spectra Test

XAFS spectra at the Ni K-edge (8333 eV) was carried out on the sample at 21A X-ray nanodiffraction beamline of Taiwan Photon Source (TPS), National Synchrotron Radiation Research Center (NSRRC). This beamline adopted 4-bounce channel-cut Si (111) monochromator for mono-beam X-ray nanodiffraction and X-ray absorption spectroscopy. The end-station equipped with three ionization chambers and S3 Lytle/SDD detector after the focusing position of KB mirror for transmission and fluorescence mode X-ray absorption spectroscopy. The photon flux on the sample is range from 1×10^{11}~3×10^{9} photon/sec for X-ray energy from 6-27 keV. The data of XANES and EXAFS were analyzed by Athena software [S6]. The Matlab source code of Wavelet transform (WT) referred Muñoz M. and co-workers' code [S7, S8].

Supplementary Figures and Tables

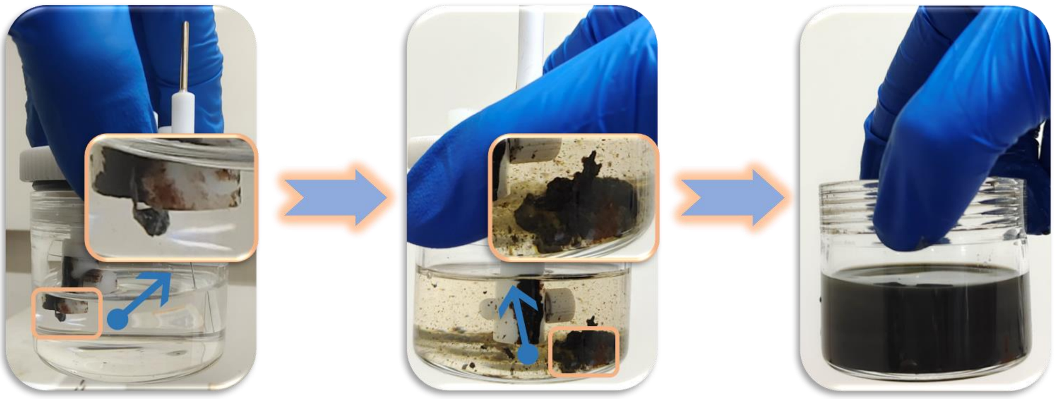

Fig. S1 Electrochemical exfoliation procedure from bulk BP to BP NSs

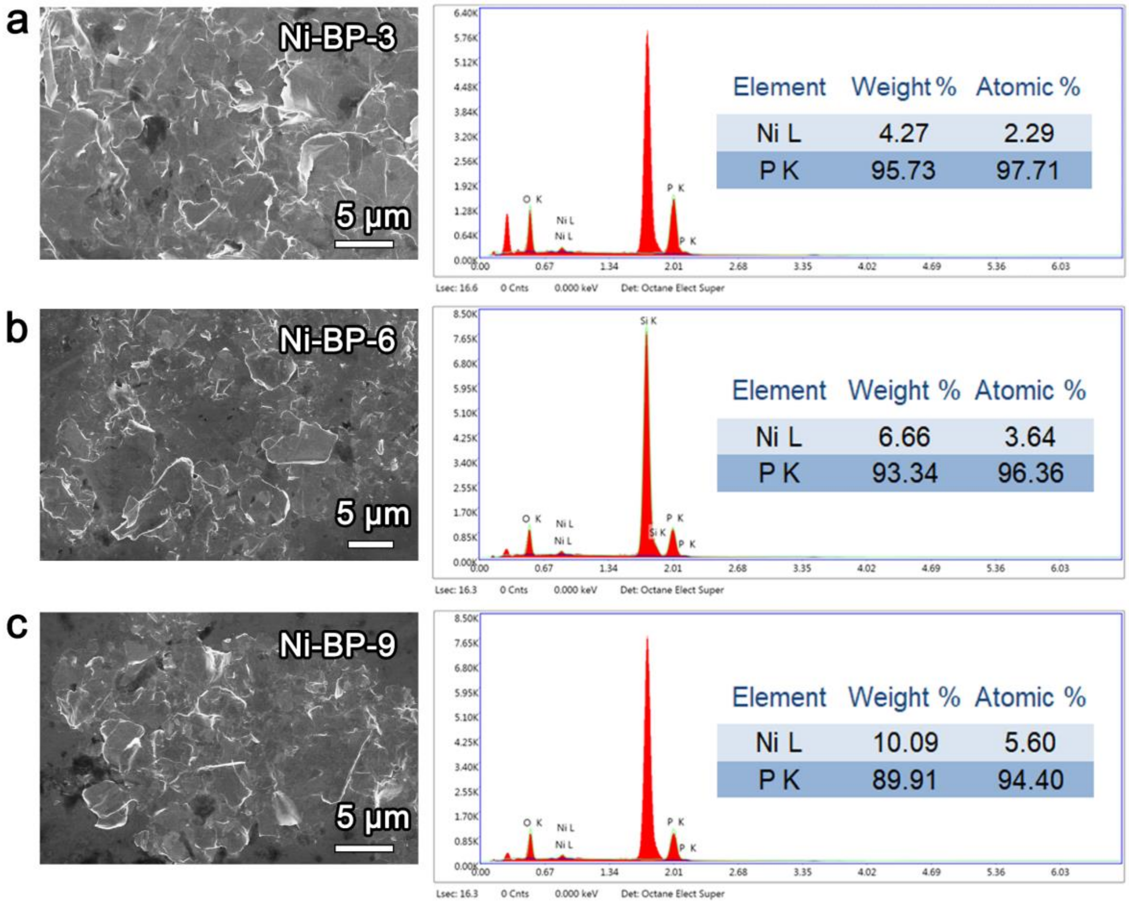

Fig. S2 SEM images: (**a**) Ni-BP-3, (**b**) Ni-BP-6, (**c**) Ni-BP-9

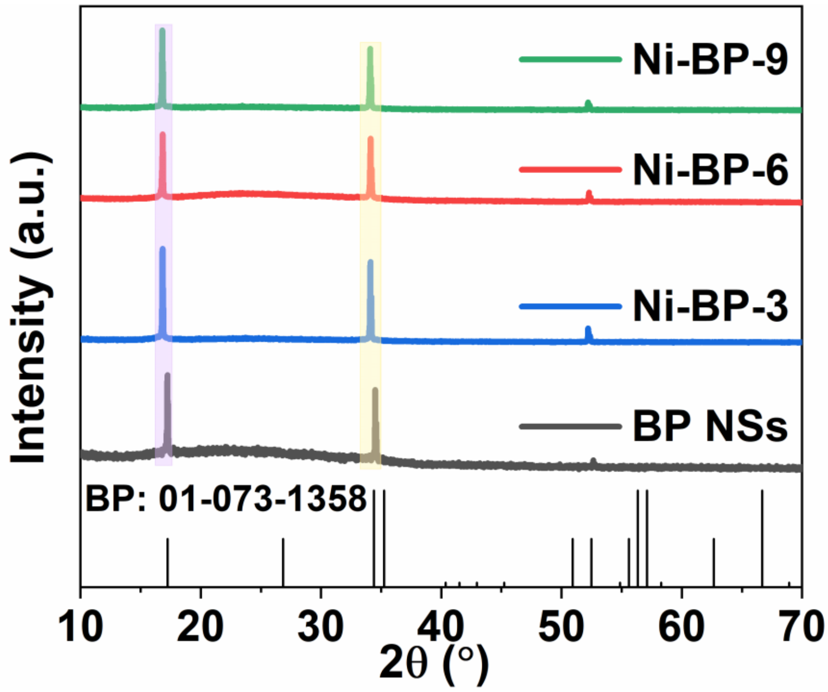

Fig. S3 XRD patterns of different samples

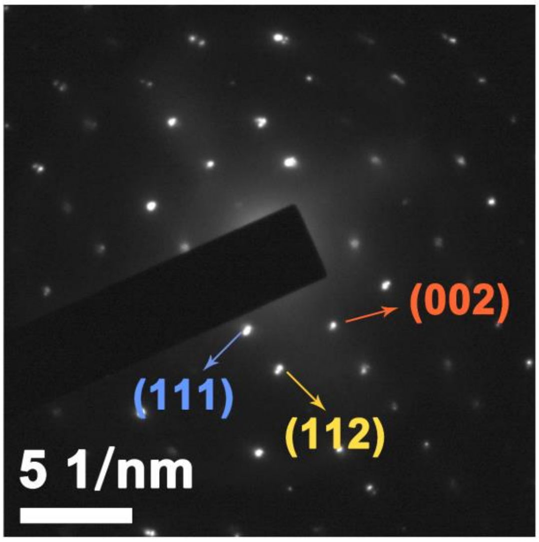

Fig. S4 The SAED pattern of BP NSs

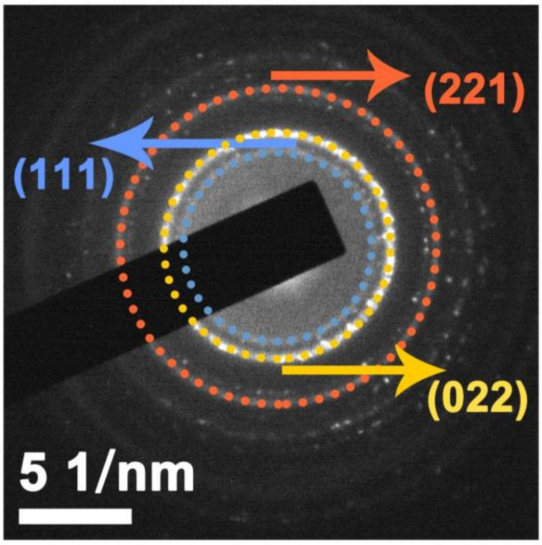

Fig. S5 The SAED pattern of Ni-BP-6

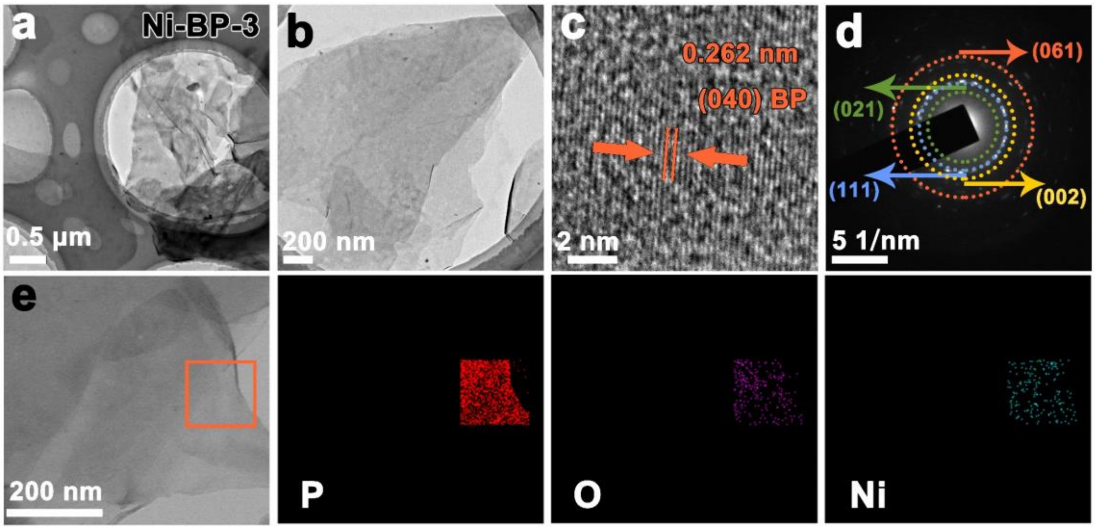

Fig. S6 TEM characterizations of Ni-BP-3: (**a, b**) TEM images, (**c**) HRTEM image, (**d**) SAED pattern, and (**e**) EDS mapping images

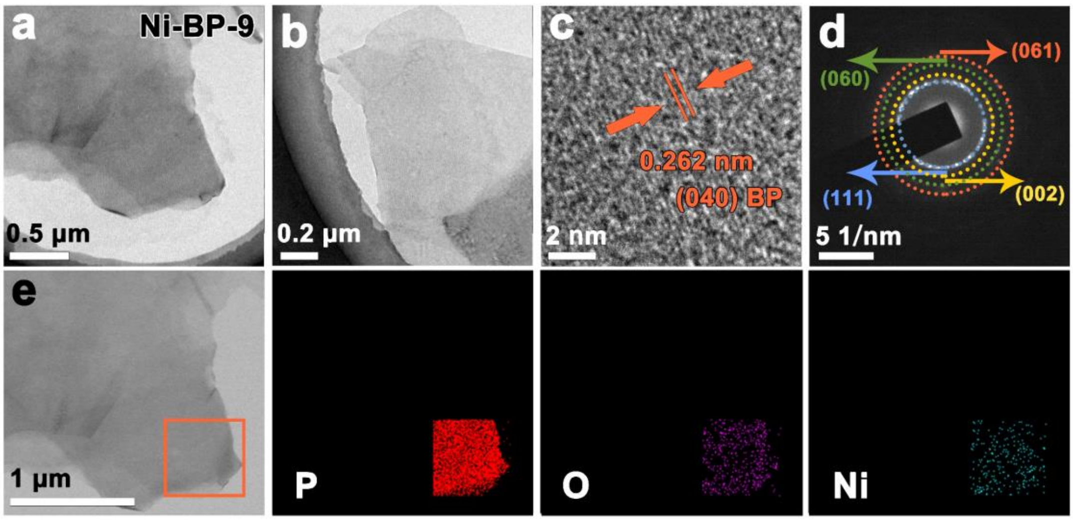

Fig. S7 TEM characterizations of Ni-BP-9: (**a, b**) TEM images, (**c**) HRTEM image, (**d**) SAED pattern, and (**e**) EDS mapping images

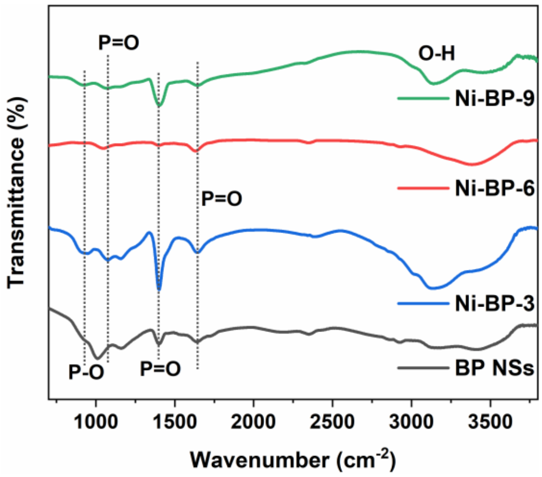

Fig. S8 FTIR spectra of samples

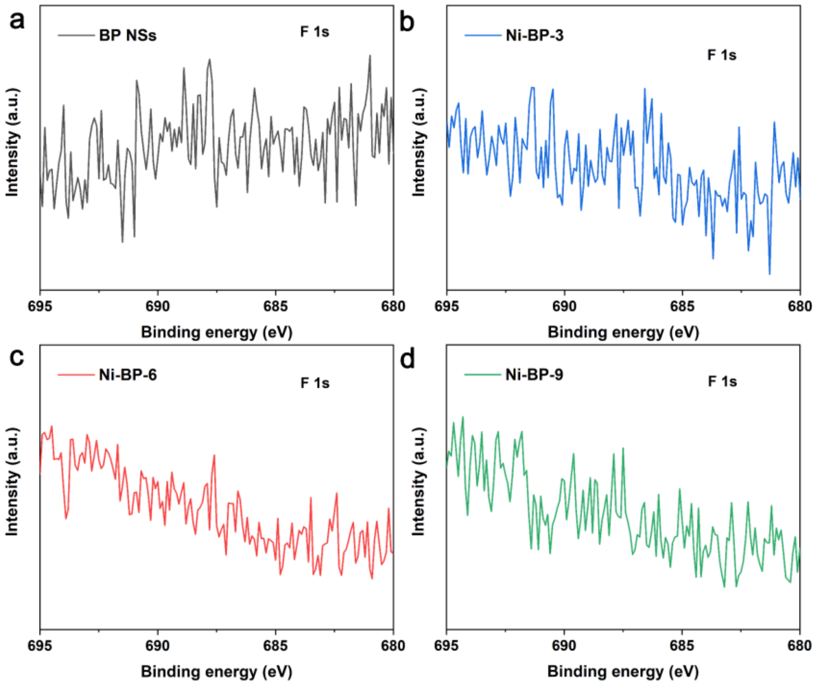

Fig. S9 F 1s XPS profiles of (**a**) BP NSs, (**b**) Ni-BP-3, (**c**) Ni-BP-6, (**d**) Ni-BP-9

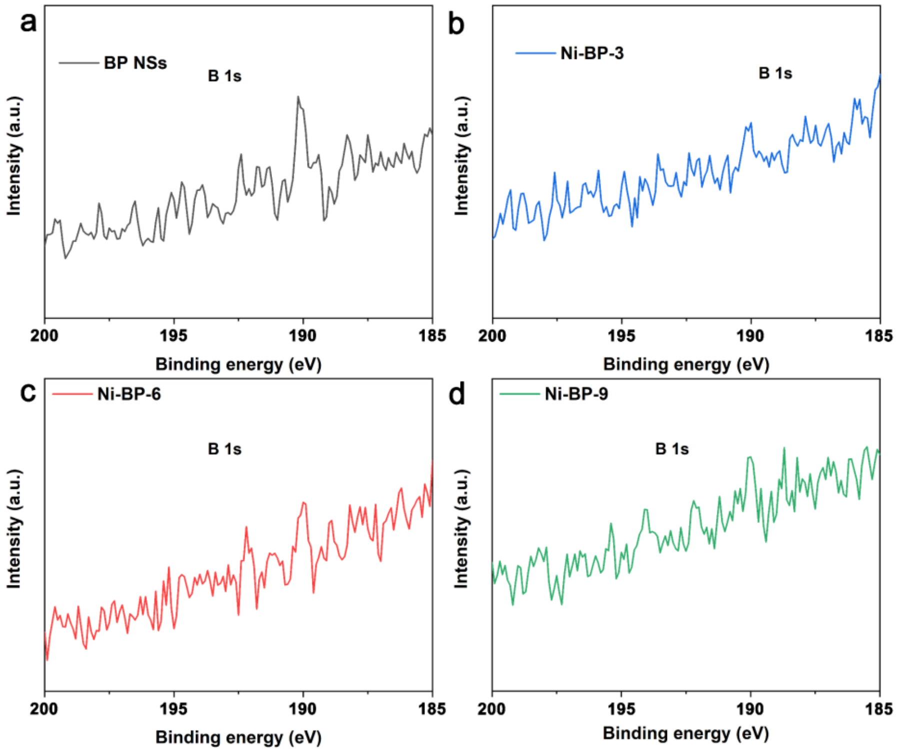

Fig. S10 B 1s XPS profiles of (**a**) BP NSs, (**b**) Ni-BP-3, (**c**) Ni-BP-6, (**d**) Ni-BP-9

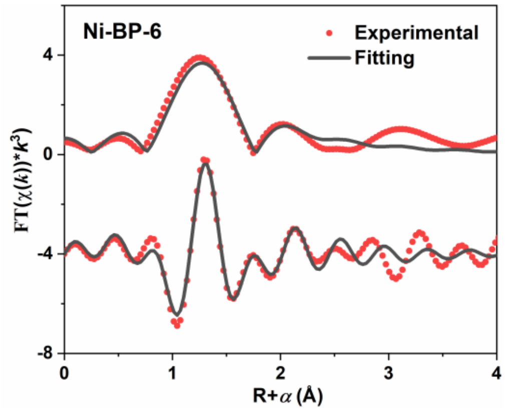

Fig. S11 Ni K-edge EXAFS (points) and curvefit (line) for Ni-BP-6, shown in R-space (FT magnitude and imaginary component). The data are k^3-weighted and not phase-corrected. Note: the detailed fitting parameters are given in Table S4.

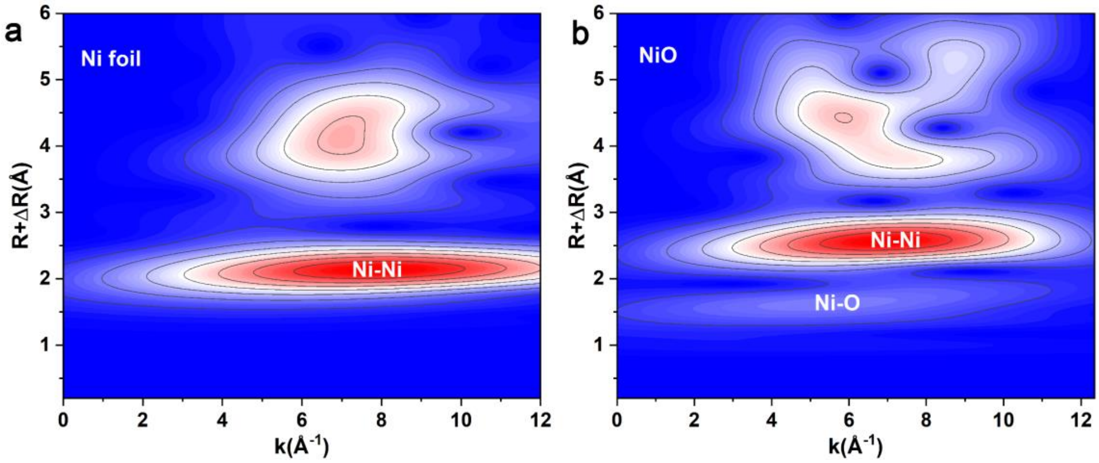

Fig. S12 Wavelet transform for the k^3-weighted EXAFS signals: (**a**) Ni foil and (**b**) NiO

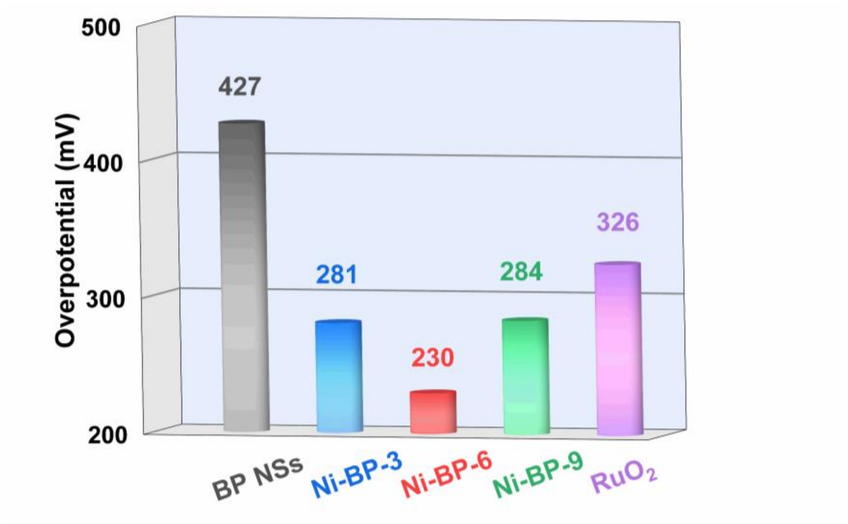

Fig. S13 Bar plots of OER overpotentials at 10 mA cm^{-2} of different electrocatalysts

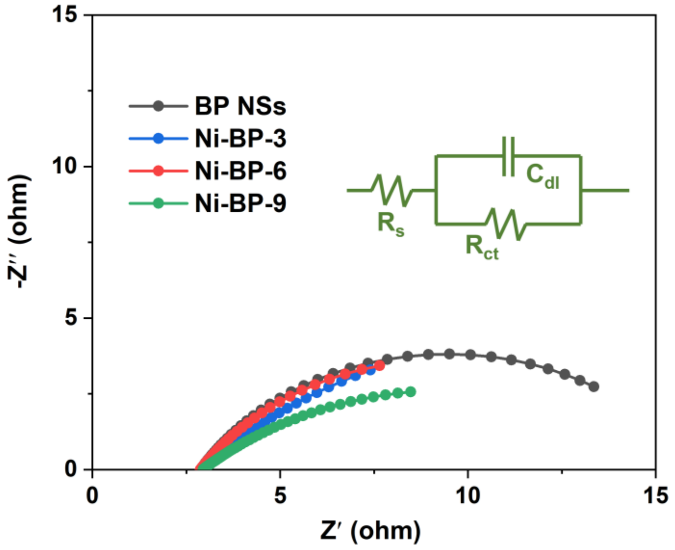

Fig. S14 The electrochemical impedance spectra of BP NSs, Ni-BP-3, Ni-BP-6, and Ni-BP-9

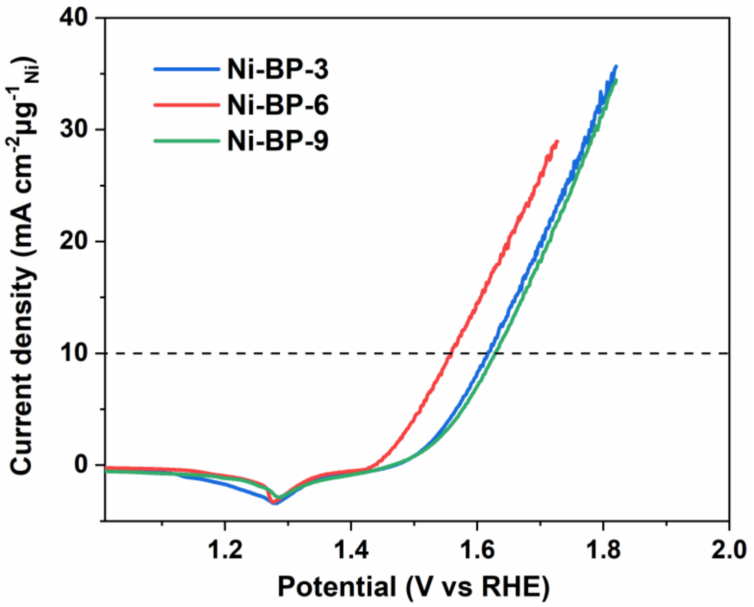

Fig. S15 LSV curves of OER catalysts normalized by Ni loading on electrodes

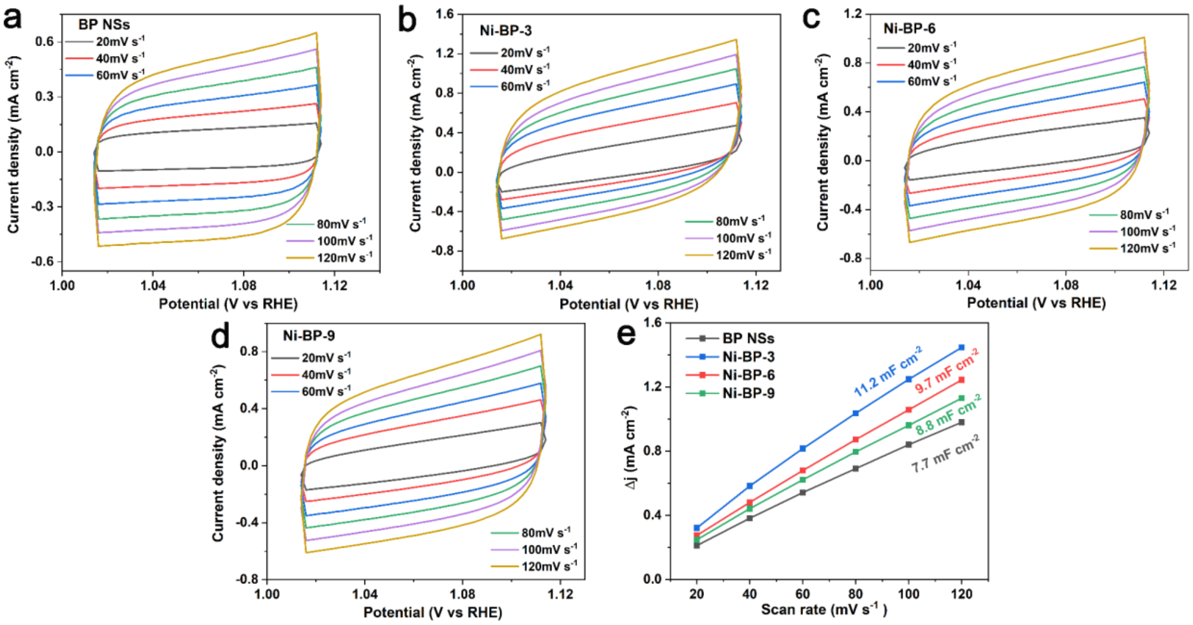

Fig. S16 CV curves for OER ECSA calculations of (**a**) BP NSs, (**b**) Ni-BP-3, (**c**) Ni-BP-6, (**d**) Ni-BP-9. (**e**) The double layer capacitance of catalysts

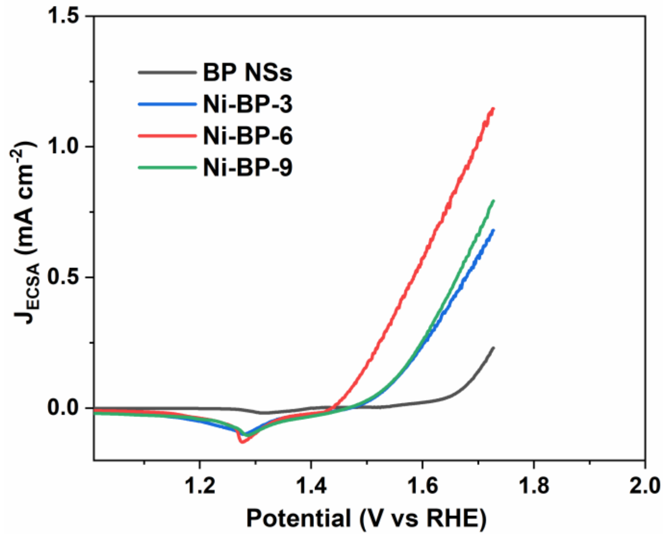

Fig. S17 ECSA-normalized LSV curves of OER catalysts

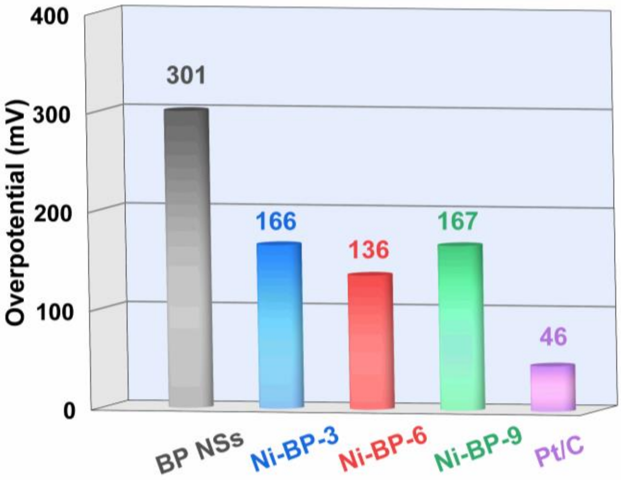

Fig. S18 Bar plots of HER overpotentials at 10 mA cm^{-2} of different electrocatalysts

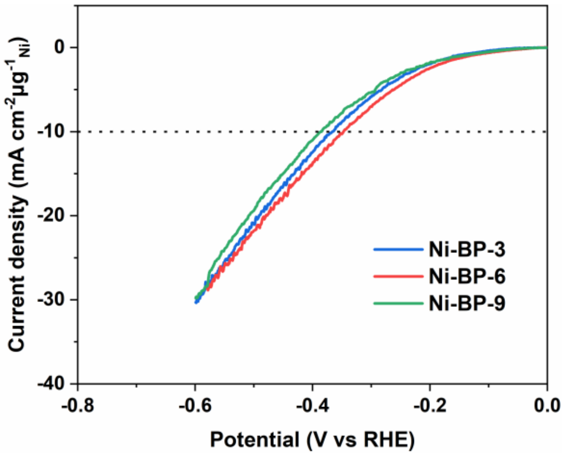

Fig. S19 LSV curves of HER catalysts normalized by Ni loading on electrodes

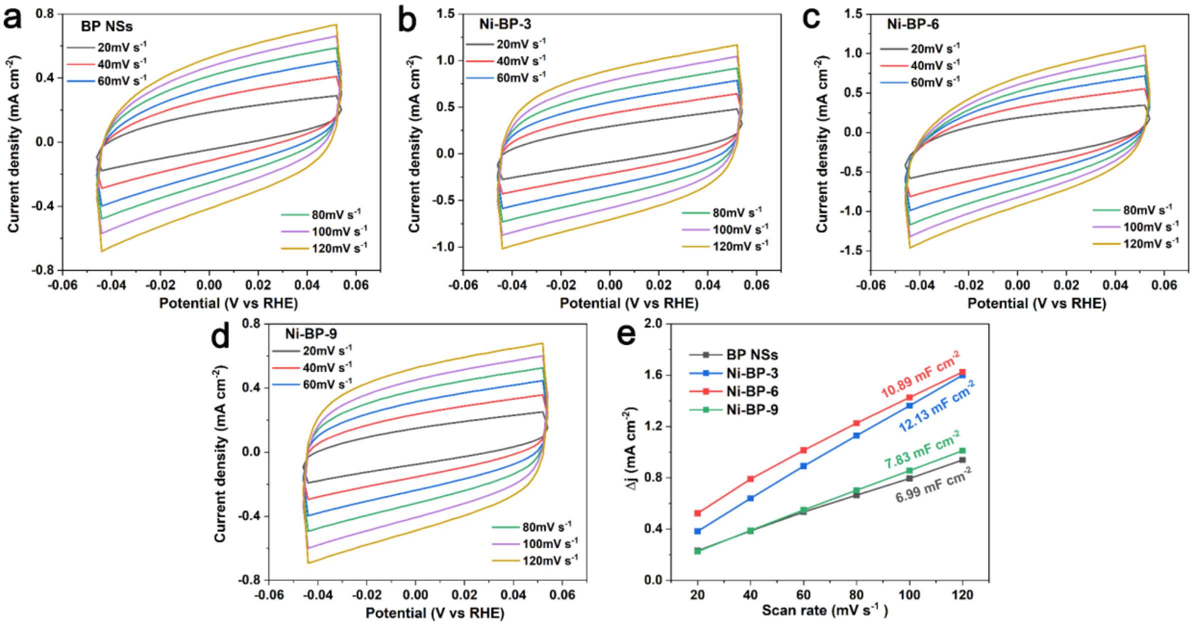

Fig. S20 CV curves for HER ECSA calculations of (**a**) BP NSs, (**b**) Ni-BP-3, (**c**) Ni-BP-6, (**d**) Ni-BP-9. (**e**)The double layer capacitance of catalysts

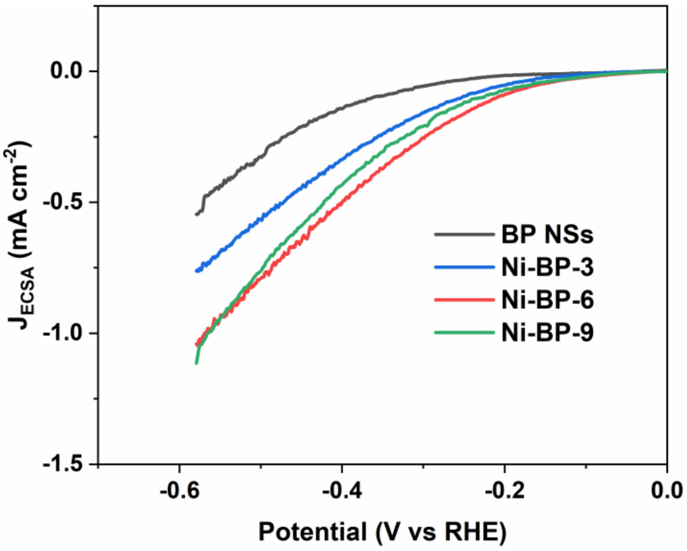

Fig. S21 ECSA-normalized LSV curves of HER catalysts

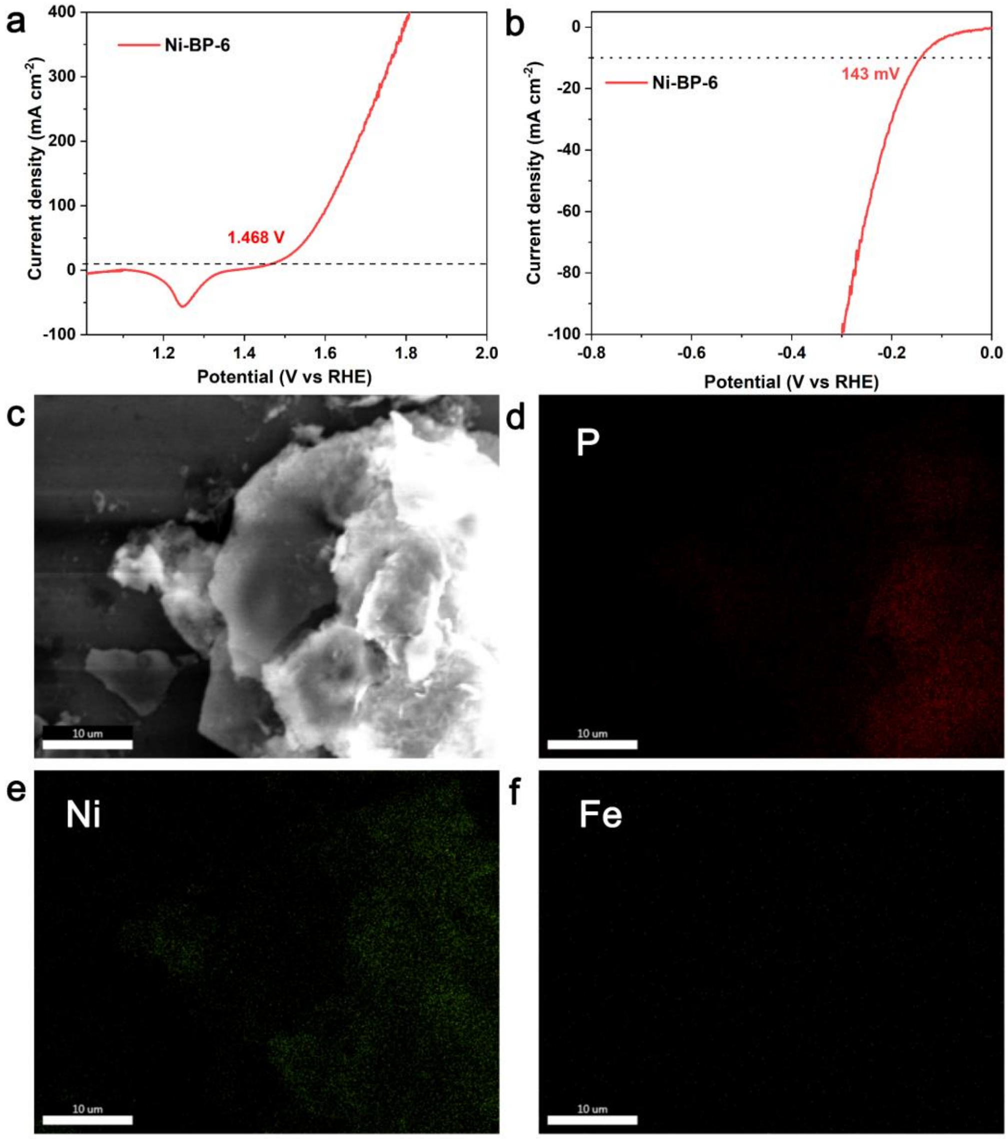

Fig. S22 (**a**) OER and (**b**) HER LSV curves of Ni-BP-6 in 1.0 M KOH (prepared by the 99.99% KOH chemical); (**c**) SEM and (**d-f**) EDS elemental mapping images of Ni-BP-6 catalyst after OER test

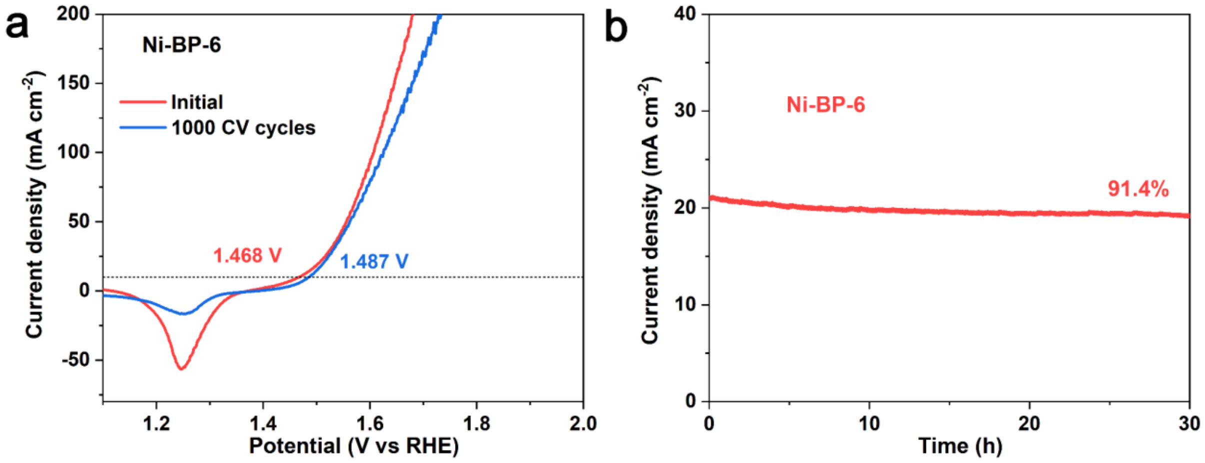

Fig. S23 (**a**) The OER LSV curve before and after 1000 CV cycles. (**b**) The OER i-t curve of Ni-BP-6 in 1.0 M KOH (prepared by the 99.99% KOH chemical)

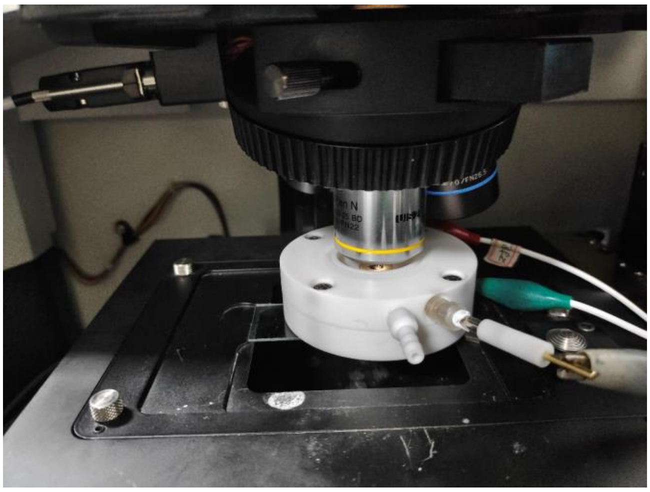

Fig. S24 Digital picture of the actual operation of the *in-situ* Raman instrument

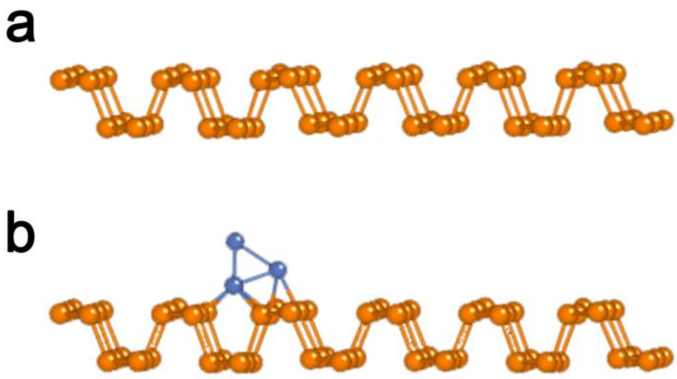

Fig. S25 DFT calculation models of the catalysts. (**a**) BP NSs. (**b**) Ni-BP-6. The brown and blue atoms represent P and Ni, respectively

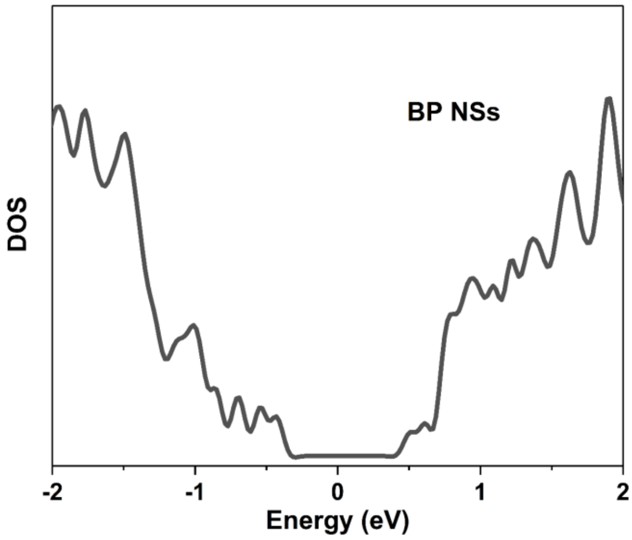

Fig. S26 DOS of BP NSs

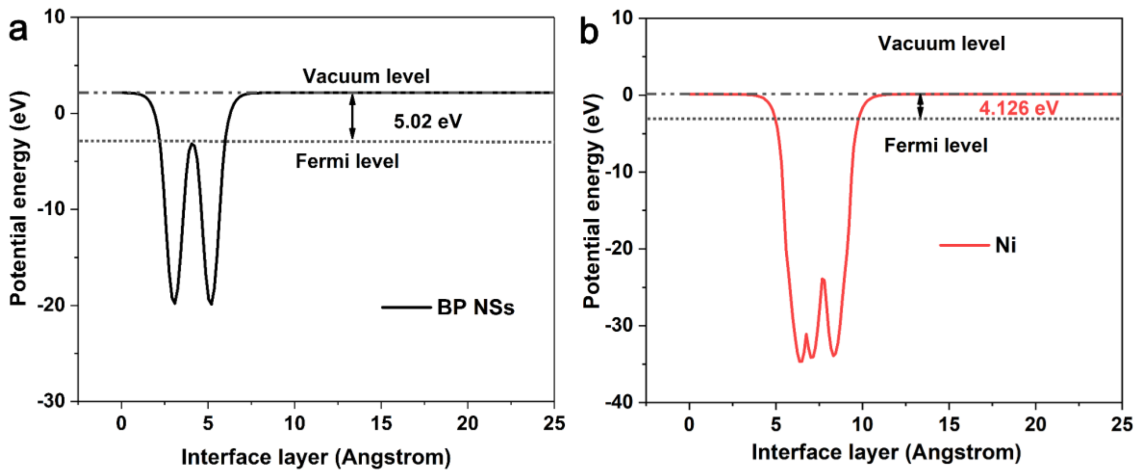

Fig. S27 The work functions of (**a**) BP NSs. (**b**) Ni4

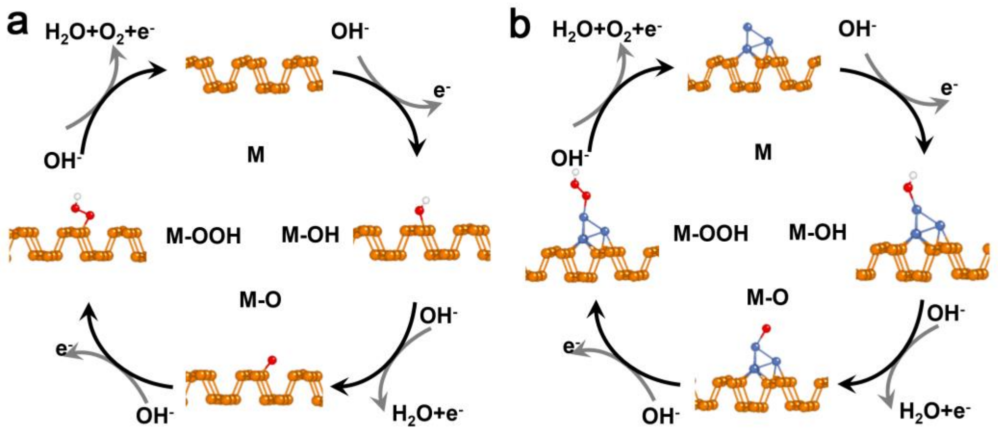

Fig. S28 The OER catalytic mechanisms of (**a**) BP NSs and (**b**) Ni-BP-6. where M means the active centers of catalyst

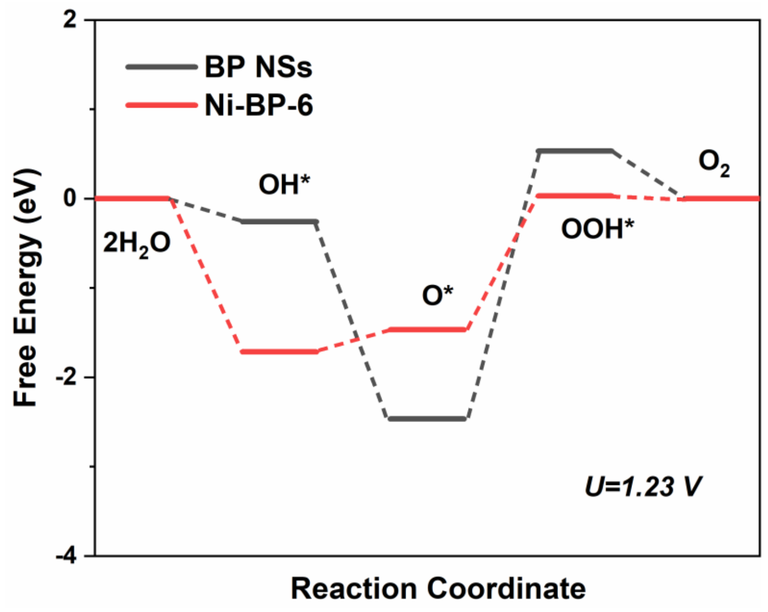

Fig. S29 OER free energy diagram on BP NSs and Ni-BP-6 at U=1.23V

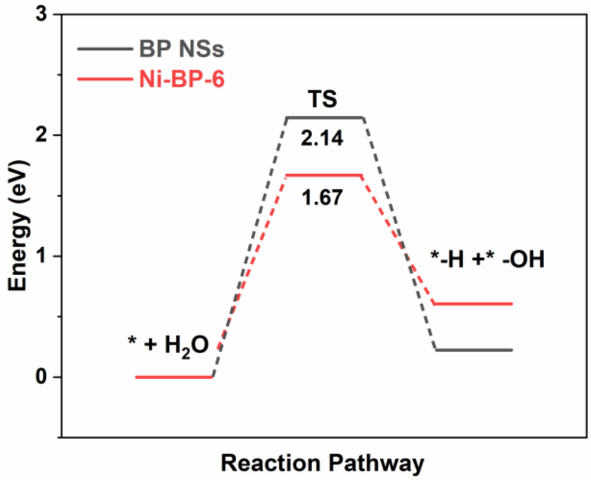

Fig. S30 Reaction energy diagram of water dissociation on BP NSs and Ni-BP-6. The initial state (H_2O), the transition state (TS) and the final state (H+OH) are indicated in the diagram with the corresponding energy barrier on the two surfaces

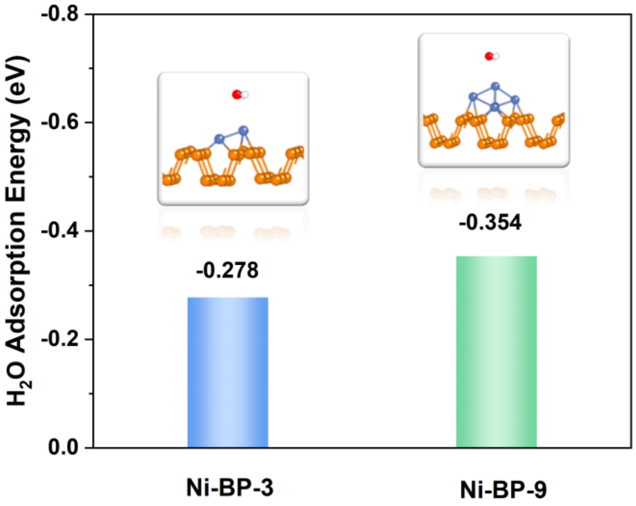

Fig. S31 H_2O adsorption model and energy of Ni-BP-3 and Ni-BP-9. The insets from left to right represent the models of Ni-BP-3 and Ni-BP-9 with the adsorption of H_2O

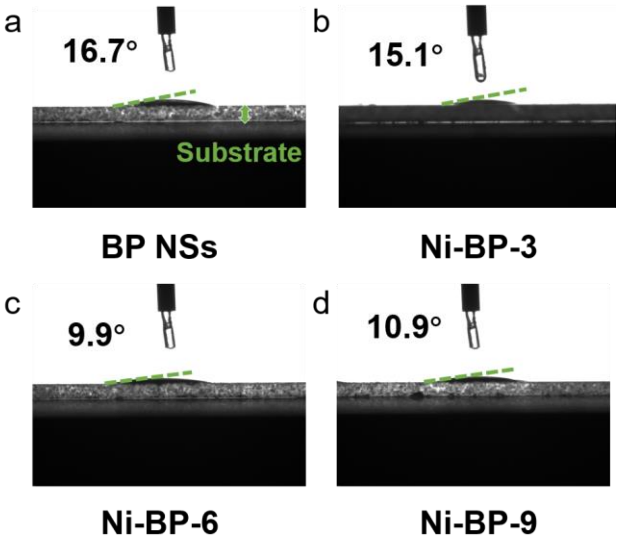

Fig. S32 Optical contact angle measurements of (**a**) BP NSs, (**b**) Ni-BP-3, (**c**) Ni-BP-6, and (**d**) Ni-BP-9

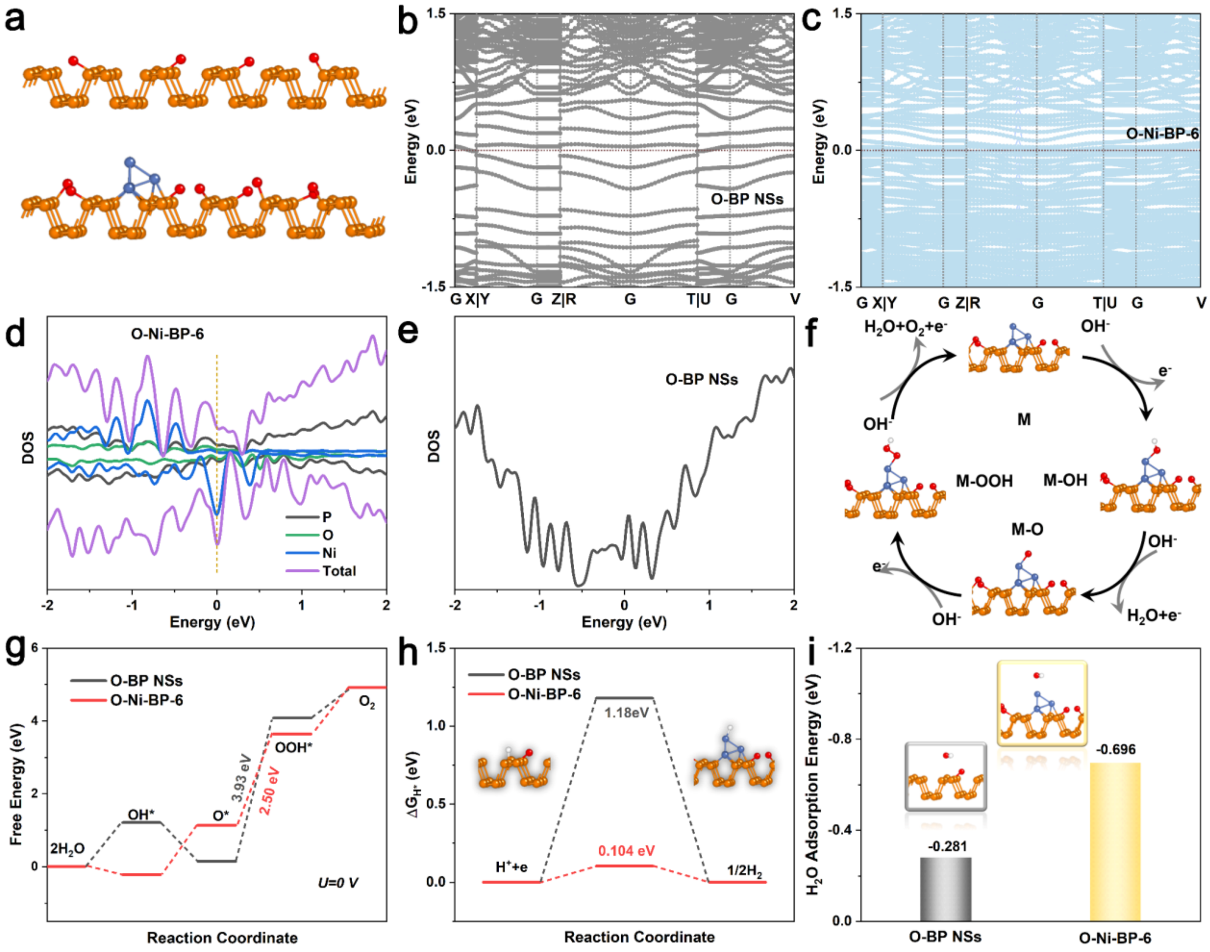

Fig. S33 Theoretically profiles for the O-BP and O-Ni-BP-6 structures. (**a**) DFT calculation models of O-BP (top) and O-Ni-BP-6 (bottom). (**b**) The band structure of O-BP. (**c**) The band structure of O-Ni-BP-6. (**d**) The DOS of O-BP. (**e**) The DOS of O-Ni-BP-6. (**f**) The OER catalytic mechanism of O-Ni-BP-6. where M means the active centers of catalyst. (**g**) The free energy diagram of the OER process on O-BP and O-Ni-BP-6 at U=0 V. (**h**) Hydrogen adsorption Gibbs free energy of O-BP and O-Ni-BP-6. The insets from left to right show the O-BP and O-Ni-BP-6 with the adsorption of hydrogen. (**i**) H_2O adsorption model and energy of O-BP NSs and O-Ni-BP-6. The insets from left to right show the O-BP NSs and O-Ni-BP-6 with the adsorption of H_2O

Table S1 ICP-MS results for Ni and P contents in Ni-BP-3, Ni-BP-6, and Ni-BP-9

**Sample**	**Ni (μg/mL)**	**P (μg/mL)**	**Weight ratios: M_{Ni}:M_{Ni+P}**
**Ni-BP-3**	6.7	978.0	0.68 *wt*%
**Ni-BP-6**	14.8	967.3	1.5 *wt*%
**Ni-BP-9**	19.6	960.2	2.0 *wt*%

Table S2 The XPS P 2p peak positions of BP NSs, Ni-BP-3, Ni-BP-6, Ni-BP-9, and Ni-BP-6-after stability testing

Sample	P-O peak (eV)	$P_{1/2}$ peak (eV)	$P_{3/2}$ peak (eV)
**BP NSs**	134.22	130.65	129.81
**Ni-BP-3**	133.65	130.23	129.38
**Ni-BP-6**	133.86	130.27	129.4
**Ni-BP-9**	133.59	130.25	129.4
**Ni-BP-6-after stability**	133.43	130.95	130.1

Table S3 The XPS Ni 2p peaks of Ni-BP-3, Ni-BP-6, Ni-BP-9, and Ni-BP-6-after stability testing

Sample	$Ni_{1/2}$ peak (eV)			$Ni_{3/2}$ peak (eV)		
	Sat.	Ni^{3+}	Ni^{2+}	Sat.	Ni^{3+}	Ni^{2+}
**Ni-BP-3**	878.9	-	874.0	860.6	-	856.3
**Ni-BP-6**	879.9	-	874.7	861.6	-	857.0
**Ni-BP-9**	879.0	-	874.1	860.3	-	856.3
**Ni-BP-6-after stability**	881.7	878.3	874.0	863.2	860.6	856.3

Table S4 Curvefit parameters[a] for Ni K-edge EXAFS for the Ni-BP-6 catalyst

Path	N	R / Å	σ^2 / Å^2
**Ni-P**	2[b]	2.27	0.0042
**Ni-Ni**	3[b]	2.46	0.0048

[a]S_0^2 was fitting as 0.78 for Ni K-edge. The inner potential correction (ΔE_0) are -7.71 eV. The number of variable parameters is 5, out of a total 7.4 independent data points.

[b]These coordination were constrained as N(Ni-P) = 2 and N(Ni-Ni) = 3 based on the Ni-BP-6 structure of DFT calculation.

Table S5 P and Ni contents in the Ni-BP-6 and in the electrolyte after i-t test by ICP-MS

Element	Concentration	Electrolyte volume	Total content
**P in electrolyte**	0.2963 μg mL^{-1}	100 mL	0.02963 mg
**Ni in electrolyte**	2.314 ng mL^{-1}	100 mL	0.2314 μg

Table S6 Comparison of OWS catalytic performances and stability at 10 mA cm^{-2} in 1.0 M KOH for Ni-BP-6‖Ni-BP-6 with other reported OWS electrocatalysts

Electrocatalysts	Current density (mA cm^{-2})	Potential	Stability (hour)	Refs.
**Ni-BP-6**	10	1.605 V	50	This work
**Pt/C‖RuO_2**	10	1.621 V	10	
**O-vac. $NiCo_2O_4$**	10	1.61 V	50	[S9]
**FeP**	10	1.62 V	28@20mA cm^{-2}	[S10]
**BP@FeCoMOF**	10	1.63 V	10@1.70 V	[S11]
**$ZnCo_2S_4$**	10	1.66 V	20	[S12]
**NiCoSe|S/BP**	10	1.67 V	10	[S13]
**MoP@Ni_3P**	10	1.67 V	45@1.65 V	[S14]
**Co-Mo_2C-CNx-2**	10	1.68 V	20@1.69 V	[S15]
**FeCo/Co_2P**	10	1.68 V	~11.1	[S16]
**NiCoO2@NiCo**	10	1.688 V	12	[S17]

Note: O-vancancy (O-vac.).

Table S7 Fe impurity content in different KOH chemicals by ICP-MS

Purity of 1 M KOH	Concentration	Electrolyte volume	Total content
**95%**	0.118 μg mL^{-1}	100 mL	11.8 μg
**99.99%**	0.029 μg mL^{-1}	100 mL	2.9 μg

Note: 5.6 g of KOH is required to configure 100 mL of 1 M KOH solution. According to Table S7, Fe impurities account for 0.21% in 95% KOH and 0.052% in 99.99% KOH.

Table S8 The OER step enegies of BP NSs

*	E(Surf+*) (eV)	Energy correction (eV)
**Slab**	-385.74509	0
**-OH**	-395.87023	0.2759
**-O**	-393.18567	0.015934
**-OOH**	-400.05989	0.297697
**Slab**	-385.74509	0

Table S9 The OER step enegies of Ni-BP-6

*	E(Surf+*) (eV)	Energy correction (eV)
**Slab**	-403.85336	0
**-OH**	-415.43564	0.275575
**-O**	-410.26542	-0.016153
**-OOH**	-418.64374	0.270674
**Slab**	-193.1032	0

Table S10 The ΔG_{H^*} of catalysts

Sample	E(Surf+H*) (eV)	E(Surf) (eV)	E(H_2) (eV)	ΔG_{H^*} (eV)
**BP NSs**	-383.375	-385.745	-6.8	1.27
**Ni-BP-6**	-400.346	-403.853	-6.8	0.13

Table S11 H_2O molecule adsorption energy on the different surface

Sample	E(Surf+H_2O^*) (eV)	E(Surf) (eV)	E(H_2O) (eV)	ΔE (eV)
**BP NSs**	-400.10009	-385.74509	-14.22	-0.135
**Ni-BP-3**	-409.37793	-394.87993	-14.22	-0.278
**Ni-BP-6**	-418.56836	-403.85336	-14.22	-0.495
**Ni-BP-9**	-424.25443	-409.68043	-14.22	-0.354

Supplementary References

[S1] D. S. Hongzhiwei Technology, Version 2021A, China. **Available:** https://iresearch.net.cn/cloudSoftware (2021).

[S2] P. E. Blöchl, Projector augmented-wave method. Phys. Rev. B **50**(24), 17953-17979 (1994). https://doi.org/10.1103/PhysRevB.50.17953

[S3] G. Kresse, J. Furthmüller, Efficient iterative schemes for ab initio total-energy calculations using a plane-wave basis set. Phys. Rev. B **54**(16), 11169-11186 (1996). https://doi.org/10.1103/PhysRevB.54.11169

[S4] J. P. Perdew, K. Burke, M. Ernzerhof, Generalized gradient approximation made simple. Phys. Rev. Lett. **77**(18), 3865-3868 (1996). https://doi.org/10.1103/PhysRevLett.77.3865

[S5] H. J. Monkhorst, J. D. Pack, Special points for Brillouin-zone integrations. Phys. Rev. B **13**(12), 5188-5192 (1976). https://doi.org/10.1103/PhysRevB.13.5188

[S6] B. Ravel, M. Newville, ATHENA, ARTEMIS, HEPHAESTUS: data analysis for X-ray absorption spectroscopy using IFEFFIT. J. Synchrotron Rad. **12**(4), 537-541 (2005). https://doi.org/10.1107/S0909049505012719

[S7] M. Muñoz, P. Argoul, F. o. Farges, Continuous cauchy wavelet transform analyses of EXAFS spectra: a qualitative approach. Am. Mineral. **88**(4), 694-700 (2003). https://doi.org/10.2138/am-2003-0423

[S8] M. Muñoz, F. Farges, P. Argoul, Continuous cauchy wavelet transform of XAFS spectra. Phys. Scr. **2005**(T115), 221 (2005). https://doi.org/10.1238/Physica.Topical.115a00221

[S9] S. Peng, F. Gong, L. Li, D. Yu, D. Ji et al., Necklace-like multishelled hollow spinel oxides with oxygen vacancies for efficient water electrolysis. J. Am. Chem. Soc. **140**(42), 13644-13653 (2018). https://doi.org/10.1021/jacs.8b05134

[S10] G. Liu, Y. Wu, R. Yao, F. Zhao, Q. Zhao et al., Amorphous iron-nickel phosphide nanocone arrays as efficient bifunctional electrodes for overall water splitting. Green

Energy Environ. **6**(4), 496-505 (2021). https://doi.org/10.1016/j.gee.2020.05.009

[S11] K. Ge, Y. Zhang, Y. Zhao, Z. Zhang, S. Wang et al., Room temperature preparation of two-dimensional black phosphorus@metal organic framework heterojunctions and their efficient overall water-splitting electrocatalytic reactions. ACS Appl. Mater. Interfaces **14**(27), 31502-31509 (2022). https://doi.org/10.1021/acsami.2c09335

[S12] G. Song, Z. Wang, J. Sun, J. Sun, D. Yuan et al., $ZnCo_2S_4$ nanosheet array anchored on nickel foam as electrocatalyst for electrochemical water splitting. Electrochem. Commun. **105**, 106487 (2019). https://doi.org/10.1016/j.elecom.2019.106487

[S13] T. Liang, S. Lenus, Y. Liu, Y. Chen, T. Sakthivel et al., Interface and M^{3+}/M^{2+} valence dual-engineering on nickel cobalt sulfoselenide/black phosphorus heterostructure for efficient water splitting electrocatalysis. Energy Environ. Mater. **6**(2), e12332 (2023). https://doi.org/10.1002/eem2.12332

[S14] F. Wang, J. Chen, X. Qi, H. Yang, H. Jiang et al., Increased nucleation sites in nickel foam for the synthesis of MoP@Ni_3P/NF nanosheets for bifunctional water splitting. Appl. Surf. Sci. **481**, 1403-1411 (2019). https://doi.org/10.1016/j.apsusc.2019.03.200

[S15] P. Zhang, Y. Liu, T. Liang, E. H. Ang, X. Zhang et al., Nitrogen-doped carbon wrapped Co-Mo_2C dual Mott–Schottky nanosheets with large porosity for efficient water electrolysis. Appl. Catal. B-Environ. **284**, 119738 (2021). https://doi.org/10.1016/j.apcatb.2020.119738

[S16] Q. Shi, Q. Liu, Y. Ma, Z. Fang, Z. Liang et al., High-performance trifunctional electrocatalysts based on FeCo/Co_2P hybrid nanoparticles for zinc–air battery and self-powered overall water splitting. Adv. Energy Mater. **10**(10), 1903854 (2020). https://doi.org/10.1002/aenm.201903854

[S17] B. Zhang, X. Zhang, Y. Wei, L. Xia, C. Pi et al., General synthesis of NiCo alloy nanochain arrays with thin oxide coating: a highly efficient bifunctional electrocatalyst for overall water splitting. J. Alloys Compd. **797**, 1216-1223 (2019). https://doi.org/10.1016/j.jallcom.2019.05.036
